# Radiomics Analysis of Magnetic Resonance Imaging Facilitates the Identification of Preclinical Alzheimer’s Disease: An Exploratory Study

**DOI:** 10.3389/fcell.2020.605734

**Published:** 2020-12-03

**Authors:** Tao-Ran Li, Yue Wu, Juan-Juan Jiang, Hua Lin, Chun-Lei Han, Jie-Hui Jiang, Ying Han

**Affiliations:** ^1^Department of Neurology, Xuanwu Hospital of Capital Medical University, Beijing, China; ^2^Center of Alzheimer’s Disease, Beijing Institute for Brain Disorders, Beijing, China; ^3^National Clinical Research Center for Geriatric Disorders, Beijing, China; ^4^Key Laboratory of Specialty Fiber Optics and Optical Access Networks, Joint International Research Laboratory of Specialty Fiber Optics and Advanced Communication, School of Information and Communication Engineering, Shanghai University, Shanghai, China; ^5^Turku PET Centre and Turku University Hospital, Turku, Finland

**Keywords:** Alzheimer’s disease, preclinical AD, radiomics, MRI, multiparametric MRI, features, imaging biomarker, cross-validation

## Abstract

Diagnosing Alzheimer’s disease (AD) in the preclinical stage offers opportunities for early intervention; however, there is currently a lack of convenient biomarkers to facilitate the diagnosis. Using radiomics analysis, we aimed to determine whether the features extracted from multiparametric magnetic resonance imaging (MRI) can be used as potential biomarkers. This study was part of the Sino Longitudinal Study on Cognitive Decline project (NCT03370744), a prospective cohort study. All participants were cognitively healthy at baseline. Cohort 1 (*n* = 183) was divided into individuals with preclinical AD (*n* = 78) and controls (*n* = 105) using amyloid-positron emission tomography, and this cohort was used as the training dataset (80%) and validation dataset (the remaining 20%); cohort 2 (*n* = 51) was selected retrospectively and divided into “converters” and “nonconverters” according to individuals’ future cognitive status, and this cohort was used as a separate test dataset; cohort three included 37 converters (13 from the Alzheimer’s Disease Neuroimaging Initiative) and was used as another test set for independent longitudinal research. We extracted radiomics features from multiparametric MRI scans from each participant, using *t*-tests, autocorrelation tests, and three independent selection algorithms. We then established two classification models (support vector machine [SVM] and random forest [RF]) to verify the efficiency of the retained features. Five-fold cross-validation and 100 repetitions were carried out for the above process. Furthermore, the acquired stable high-frequency features were tested in cohort three by paired two-sample *t*-tests and survival analyses to identify whether their levels changed with cognitive decline and impact conversion time. The SVM and RF models both showed excellent classification efficiency, with an average accuracy of 89.7–95.9% and 87.1–90.8% in the validation set and 81.9–89.1% and 83.2–83.7% in the test set, respectively. Three stable high-frequency features were identified, all based on the structural MRI modality: the large zone high-gray-level emphasis feature of the right posterior cingulate gyrus, the variance feature of the left superior parietal gyrus, and the coarseness feature of the left posterior cingulate gyrus; their levels were correlated with amyloid-β deposition and predicted future cognitive decline (areas under the curve 0.649–0.761). In addition, levels of the variance feature at baseline decreased with cognitive decline and could affect the conversion time (*p* < 0.05). In conclusion, this exploratory study shows that the radiomics features of multiparametric MRI scans could represent potential biomarkers of preclinical AD.

## Introduction

Alzheimer’s disease (AD) is an evolving medical challenge that represents the largest unmet medical need because of its epidemiology and irreversible as well as incurable nature (Long and Holtzman, 2019; Jia et al., 2020). A series of disappointing large-scale clinical trials in symptomatic patients have resulted in clinical consensus that efforts should move forward to the early stages of the disease (Sperling et al., 2011b; Golde et al., 2018). According to the latest National Institute on Ageing–Alzheimer’s Association (NIA-AA) diagnostic framework, cognitively healthy individuals with brain amyloid-β (Aβ) deposition have already entered the irreversible Alzheimer’s continuum (Jack et al., 2018) and have a higher risk of developing cognitive and functional decline (Papp et al., 2017; Insel et al., 2018). These individuals can be defined as preclinical AD patients. The accurate ultra-early diagnosis of the preclinical stage of AD exactly provides a window of opportunity for intervention and is thus of great clinical importance and being the first imperative.

As Aβ deposition is the criterion standard for the diagnosis of preclinical AD, its detection has become a crucial issue. Currently, the internationally recognized state-of-the-art biopsy assessment for brain Aβ depends on amyloid positron emission tomography (PET) imaging and cerebrospinal fluid analysis (Jack et al., 2018); however, their popularization has been limited by cost and the invasiveness of the procedure (Li T. R. et al., 2019). Hence, there is an urgent need for a convenient and inexpensive diagnostic technique. Magnetic resonance imaging (MRI) has been widely used in the clinical evaluation of neurodegeneration and has been incorporated into the AD diagnostic framework (Jack et al., 2018); comparatively, functional MRI (fMRI) and diffusion tensor imaging (DTI) are essentially limited to scientific research. Considerable research progress has been made in the discrimination of mild cognitive impairment (MCI) and dementia through the use of these different imaging modalities (Promteangtrong et al., 2015), which have shown promise in the identification of preclinical AD; however, there is currently a lack of diagnostic biomarkers.

Radiomics, a method of high-dimensional minable data analysis, can quantitatively examine a large set of phenotypic features and has previously been successfully applied to the evaluation of multiparametric MRI (MPMRI) and PET as imaging biomarkers in AD (Rathore et al., 2017; Zhou et al., 2018; Li Y. et al., 2019). Many studies have shown changes in the volumetric and morphometric indices of specific brain regions, including the hippocampus, thalamus, callosum, and cingulate gyrus, in the prophase of cognitive decline (Baron et al., 2001; Thomann et al., 2006; Balthazar et al., 2009; Pedro et al., 2012; Guo et al., 2014). Recent studies of texture analysis have suggested that abnormalities of textural features occur early (Sørensen et al., 2016; Feng F. et al., 2018; Lee et al., 2020) and can also distinguish between healthy controls, AD-MCI, and AD–dementia patients based on cortical, subcortical, or whole-brain analysis (de Oliveira et al., 2011; Li et al., 2014; Chaddad and Niazi, 2018; Chaddad et al., 2018; Feng F. et al., 2018; Feng Q. et al., 2018; Luk et al., 2018; Kun et al., 2020), and their accuracy in predicting the transition from MCI to dementia is higher than that of volume reduction (Sørensen et al., 2016; Luk et al., 2018; Lee et al., 2020). Relative to controls, both AD-MCI and AD–dementia patients showed widespread changes in multiple indices of DTI (Alves et al., 2012; Gyebnár et al., 2018). These findings highlight the potential use of MPMRI radiomics analysis as a measure of neurodegenerative processes in AD, which may contain unique information about changes at the microscopic level that can occur before changes at the macroscopic level, such as atrophy. However, to the best of our knowledge, no such studies focusing on preclinical AD have been previously reported. Deep learning is another effective classification method, but it requires a large number of image datasets, and clinicians cannot obtain interpretable features as imaging biomarkers (Yamanakkanavar et al., 2020); thus, we did not utilize this methodology.

With this study, we aimed to (a) explore novel imaging biomarkers based on radiomics analysis of MPMRI [structural MRI (sMRI), fMRI, and DTI]; and (b) employ classification models to discriminate preclinical AD based on radiomics features.

## Materials and Methods

### Study Design

**FIGURE 1:**
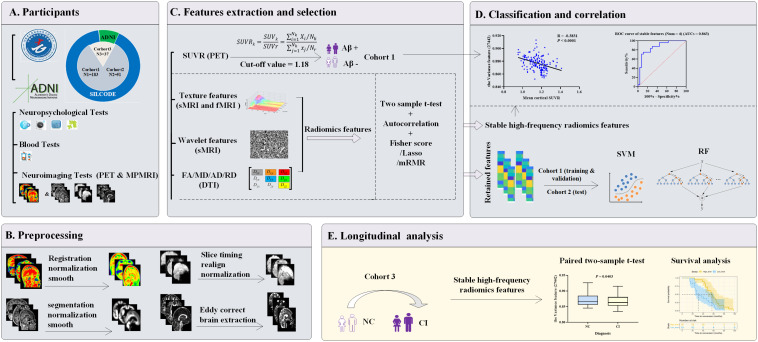
Workflow diagram. **(A)** Three cohorts were enrolled. Cohort 1 (*n* = 183) and cohort 2 (*n* = 51) were both from the SILCODE project and divided qualitatively by Aβ status or future cognitive outcomes, respectively. Cohort 3 (*n* = 37) included 24 “converters” from SILCODE and 13 from the ADNI. All participants were cognitively healthy at baseline and were evaluated in a standardized protocol. **(B)** Preprocessing of amyloid PET and MPMRI was performed for each modality. **(C)** Radiomics features were extracted from each modality, and a novel method characterized by the combination of function perturbations (*t*-test, autocorrelation test, and three independent algorithms) and sample perturbations (five-fold cross-validation and 100 repetitions) was performed to select features from the training dataset (80% of cohort 1). **(D)** Retained features were incorporated into classification models and verified in the validation (the remaining 20% of cohort 1) and test dataset (cohort 2), respectively. Furthermore, during the process of selection, stable high-frequency features were identified that were undisturbed by perturbations and correlated with the SUVR values, which played a good role in predicting prospective cognitive decline (cohort 2). **(E)** Levels of stable high-frequency features were tested to determine whether they change with cognitive decline or impact the conversion time. ADNI, Alzheimer’s Disease Neuroimaging Initiative; MRI, magnetic resonance imaging; sMRI, structural MRI; fMRI, functional MRI; DTI, diffusion tensor imaging; MPMRI, multiparameter MRI; PET, positron emission tomography; FA, fractional anisotropy; MD, mean diffusivity; AD, axial diffusivity; RD, radial diffusivity; SUVR, standard uptake value ratio; Aβ, amyloid β; Lasso, least absolute shrinkage and selection operator; mRMR, max-relevance and min-redundancy; SVM, support vector machine; RF, random forest; ROC, receiver operating characteristic; AUCs, areas under curve; NC, normal control; CI, cognitive impairment.

The comprehensive workflow is shown in Figure 1, including establishment of the cohorts (A), preprocessing of images (B), extraction and selection of radiomics features (C), model establishment, classification experiments, correlation analysis (D), and longitudinal studies of typical features (E).

### Participants

The study was part of the Sino Longitudinal Study on Cognitive Decline (SILCODE), an ongoing prospective cohort study (ClinicalTrials.gov identifier: NCT03370744; the protocol can be accessed at ClinicalTrials.gov) (Li X. et al., 2019), which centers on Xuanwu Hospital in cooperation with an alliance of 94 hospitals from 50 cities in China. The SILCODE project is a constellation of interconnected substudies, and one of its aims is to assess the diagnostic application of imaging in different stages of the cognitive continuum. Therefore, baseline standardized clinical evaluation and MPMRI were offered to all participants, resulting in the enrollment of 1,594 individuals with different diagnoses and ranging from being cognitively unimpaired to a confirmed diagnosis of dementia. In this study, we established three cohorts from the database and the Alzheimer’s Disease Neuroimaging Initiative (ADNI,^1^) with high selectivity. In cohort 1, 183 cognitively healthy participants with amyloid-PET imaging were recruited between July 2016 and November 2018 sequentially, all from the SILCODE project (Supplementary Figure 1A). In cohort 2, 51 participants were included; they participated in the SILCODE project between December 2009 and December 2015, were selected retrospectively, and were interviewed every 10–15 months until the end of 2019, with 24 later experiencing cognitive decline and 27 remaining healthy (Supplementary Figure 1B). In cohort 3, the 24 “converters” from cohort 2 and an additional 13 individuals from ADNI were included; they all underwent MPMRI examinations at baseline and also when cognitive deterioration was first identified. The entry criteria for healthy individuals have been described previously (Chen et al., 2019; Li X. et al., 2019). The diagnosis of dementia was based on the guidelines of the NIA-AA workgroups (McKhann et al., 2011), and of MCI was based on Petersen’s criteria (before 2016) (Petersen, 2004) or a neuropsychological method (after 2016) (Bondi et al., 2014).

Participants in cohort 1 underwent a dynamic scan with Florbetapir F-18 (AV45). The whole brain voxel-wise standardized uptake value ratio (SUVR) was normalized to the whole cerebellum, representing the mean cortical SUVR. For the dichotomy, amyloid-PET positivity (that is, participants who in the preclinical stage of the Alzheimer’s continuum) was defined *a priori* with the established cutoff of >1.18 (Fakhry-Darian et al., 2019). The results for each participant were confirmed by two senior radiologists who were blinded to any clinical information and made positive or negative judgments. If the judgment was inconsistent, a third radiologist was consulted to arbitrate in the dispute.

Informed consent was obtained from all participants. Further details regarding the rigorous evaluation of our participants are presented in Supplementary Figure 1 and Material.

### Imaging Acquisition and Preprocessing

The MRI data of participants from SILCODE project were acquired using a 3.0-T MRI scanner (Magnetom Sonata; Siemens Healthineers AG, Erlangen, Germany) before 2016, or an integrated simultaneous 3.0-T time-of-flight PET/MR (SIGNA; GE Healthcare, Chicago, IL, United States) after that time point. Before undergoing imaging, subjects were instructed to keep their eyes closed but not fall asleep, relax their minds, and move as little as possible during imaging. Foam pads and headphones were used to minimize head movement and scanner noise. The sMRI was obtained with a magnetization prepared rapid gradient echo sequence (Siemens/GE): repetition time (TR) = 1,900 ms/6.9 ms, echo time (TE) = 2.2 ms/2.98 ms, slice number = 176/192; fMRI was obtained with a single-shot gradient-echo echo planar imaging (EPI) sequence (Siemens/GE): TR = 2,000 ms/2,000 ms, TE = 40 ms/30 ms, slice number = 28/28; and a single-shot spin-echo diffusion-weighted EPI sequence was used for the DTI data (Siemens/GE): TR = 11,000 ms/16,500 ms, TE = 98 ms/95.6 ms, slice number = 60/75. The detailed protocols can be found in the Supplementary Material.

The images of the ADNI participants were downloaded from the ADNI database. Detailed information regarding the acquisition protocol is publicly available on the LONI website^2^.

The standardized preprocessing of amyloid PET and MPMRI has been described in previous studies (Hassan et al., 2016; Li X. et al., 2019; Tian et al., 2019). The original DICOM data were converted to the NIfTI file format by using DCM2NII^3^. We then processed the MPMRI and amyloid-PET imaging data separately for each participant. For sMRI, the images were normalized and showed a spatial resolution of 91-mm × 109-mm × 91-mm with a 2-mm × 2-mm × 2-mm voxel size after being segmented into gray matter, white matter, and cerebrospinal fluid tissues. We then smoothed them using an isotropic Gaussian smoothing kernel with a full width at half maximum of 4 mm × 4 mm × 4 mm. For fMRI, the first 10 time-point images were discarded for magnetization balance. After that, the remaining 230 time-point images were corrected and aligned to the first time-point image to correct for head movements. The resulting motion-corrected volumes were coregistered to the anatomical T1-weighted images and normalized to the Montreal Neurological Institute template, resampling to a 3-mm cube voxel resolution. For DTI, we employed the Eddy Correct tool to correct the head motion and eddy current distortions (Wang et al., 2016; Tang et al., 2017) and used the brain extraction tool to remove the nonbrain tissues of the B0 image and create the brain mask (Smith, 2002). We then adopted the DTIFIT tool to fit the diffusion tensor at each voxel and produced four parameter maps encompassing fractional anisotropy, mean diffusivity, axial diffusivity, and radial diffusivity (Basser et al., 1994). For amyloid PET, the structural images were individually registered to the averaged PET images. We then performed segmentation of all the coregistered structural images, spatially normalizing the PET images to the Montreal Neurological Institute standard space by using the forward parameters (estimated during the segmentation), and smoothed the images with an 8-mm full width at half maximum Gaussian kernel.

The sMRI and fMRI images were preprocessed using the Data Processing Assistant for resting-state fMRI (DPASF;^4^) implemented in MATLAB R2018a (MathWorks, Natick, MA, United States) (Chao-Gan and Yu-Feng, 2010). DTI was performed using the pipeline for analyzing brain diffusion images (PANDA) implemented based on the FMRIB Software Library (Smith et al., 2004); amyloid-PET data were obtained using the Statistical Parametric Mapping (SPM12;^5^) implemented in MATLAB.

### Feature Extraction

Feature extraction was performed for each modality separately. For sMRI, 43 texture features and 172 wavelet features of each region of interest (ROI; 116 in total, based on the AAL template) were extracted. For fMRI, 43 texture features of each ROI were extracted. For DTI, we calculated the white matter tracts and viewed the fractional anisotropy, mean diffusivity, axial diffusivity, and radial diffusivity as features. All extracted features were adjusted before selection using linear regression to control for the impact of age, gender, and education.

Feature extraction of the sMRI and fMRI data was performed using the Texture Toolbox from radiomics tools developed by Vallières et al. (2015)^6^ based on MATLAB; for DTI, the procedure was carried out using the PANDA (Smith et al., 2004; Cui et al., 2013). Further details are described in the Supplementary Table 1 and Material.

### Feature Dimensionality Reduction and Selection

This step was achieved using MATLAB. More specifically, we performed a five-fold cross-validation on the dataset of cohort 1; that is, the data were randomly divided into a training set (80%) and a validation set (the remaining 20%). In the training set, three steps including *t*-tests, autocorrelation tests, and three independent algorithms [Fisher score, least absolute shrinkage and selection operator (Lasso), and max-relevance and min-redundancy (mRMR)] were adopted to filter the redundant and meaningless features. The remaining features were retained and incorporated into classification models. Importantly, we repeated the above steps 100 times. More details can be found in the Supplementary Material.

In addition, for each type of the above three algorithms, we calculated the number of occurrences of each retained feature, ranging from 0 to 500. The top 10 most frequently appearing features were defined as high-frequency features, and the stable high-frequency features represented the overlaps among the three perturbations.

### Classification Experiments

The support vector machine (SVM) and random forest (RF) are both popular and mature machine learning algorithms with a solid theoretical basis (Uddin et al., 2019). Here, these two classification models were established to verify the performance of retained features in the validation (20% of cohort 1) and test set (cohort 2), respectively. The SVM model employed three different kernels: sigmoid, linear, and radial basis. Corresponding to the retained features, there were 500 permutation experiments using the Fisher score, Lasso, or mRMR algorithm. The final accuracy, sensitivity, and specificity results were presented as average values ± standard deviation (SD) for each model.

In addition, receiver operating characteristic (ROC) analyses were performed to evaluate the ability of each stable high-frequency feature in predicting prospective cognitive decline of participants in the test set, and the areas under curve (AUCs) were calculated. The analysis was performed using SPSS 13.0 software (SPSS Inc., Chicago, IL, United States). Further details are provided in the Supplementary Material.

### Longitudinal Analyses

As an independent longitudinal research study, this aspect of the study aimed to further clarify the role of the stable high-frequency features identified from the training dataset (80% of cohort 1) in another separate test dataset, that is, cohort 3. The feature extraction was identical to that mentioned above. In order to test whether the levels of stable high-frequency features changed with cognitive decline, we performed comparisons at two different time points of cognitive stages and verified whether these features affected the conversion time of individuals using survival analyses.

### Statistical Analysis

The demographic data of participants are summarized as numbers (%) or mean ± SD for categorical and continuous variables, respectively. The between-group comparisons were performed using the χ^2^ test for categorical variables or the two-sample *t*-test for continuous variables (two-tailed). A *p* < 0.05 was considered statistically significant.

In the process of dimensionality reduction, the two-sample *t*-test was two-tailed and considered significant when *p* < 0.05; for the autocorrelation test, we calculated the Pearson correlation coefficients between features and considered the paired features to have a high correlation when values in the pairwise correlation were greater than 0.8. Furthermore, in order to better understand the association between radiomics features and iconic pathological changes in AD, we created Pearson correlations to evaluate the relationship between stable high-frequency features and mean cortical SUVR values and acquired the results after adjusting for age, gender, education, and Montreal Cognitive Assessment (MoCA) scores.

In the longitudinal analyses, we mapped out the changing trajectory of each stable high-frequency feature at the individual level and performed paired two-sample *t*-tests at the group level (two-tailed, *p* < 0.05). In the survival analyses, individuals of cohort 3 were divided into two parts, the high-level group (*n* = 18) and low-level group (*n* = 19), according to the median level of each stable high-frequency feature. Subsequently, cumulative probabilities of clinical conversion by the two groups were displayed according to the Kaplan–Meier method, and the survival curves were compared between groups in a univariate analysis using the log-rank test.

These above analyses were performed in SPSS or MATLAB.

## Results

### Participants

In cohort 1,183 healthy participants were included. Their clinical and MPMRI examinations were almost continuous, and amyloid PET was performed within 3 months of the MPMRI scan. Eventually, 78 amyloid-positive and 105 negative participants were identified. Compared to the negative individuals, individuals who were positive were older (*p* = 0.039) and had a higher AV45 SUVR (*p* < 0.0001), but there were no statistical differences in the other clinical data collected (Table 1).

**TABLE 1:** Clinical characteristics of participants.

	Cohort 1			Cohort 2				Cohort 3
Group	Aβ _P (*n* = 78)	Aβ _N (*n* = 105)	*P*-value	Cog_D (*n* = 24)	Cog_M (*n* = 27)	*P*-value	ADNI (*n* = 13)	ADNI + Cog_D (*n* = 37)
Gender (male/female)	21/57	36/69	0.280	11/13	11/16	0.714	4/9	15/22
Age (y)	67.4 ± 6.0	65.6 ± 5.5	0.039*	69.5 ± 7.6	68.9 ± 7.3	0.775	74.85 ± 6.75	73.32 ± 9.06
Education	12.8 ± 3.6	12.3 ± 3.1	0.275	11.0 ± 4.8	13.0 ± 2.7	0.074	14.7 ± 1.2	12.30 ± 4.29
MoCA	26.2 ± 3.1	25.9 ± 2.8	0.486	23.5 ± 1.9 (23 Ava)	25.0 ± 3.2 (26 Ava)	0.054	23.8 ± 3.8 (12 Ava)	23.7 ± 2.7 (35 Ava)
APOE ε4	28 (77 Ava, 36.4%)	38 (36.2%)	0.981	9 (21 Ava, 42.9%)	13 (48.1%)	0.715	1 (7.7%)	10 (34 Ava, 29.4%)
SUVR	1.230 ± 0.047	1.089 ± 0.057	<0.0001**					

Cohort 1 was qualitatively divided into Aβ-P and Aβ-N groups according to participants’ SUVR (cutoff: 1.18); cohort 2 was classified by participants’ future cognitive outcomes, including Cog-D and Cog-M groups. At baseline, all participants were cognitively healthy, and we made comparisons of the clinical data between the two groups of cohorts 1 and 2. Cohort 1 was used as the training and validation dataset, and cohort 2 was used as the test set. Cohort 3 was applied to the longitudinal study, including the 24 participants from cohort 2 (Cog-D) and 13 from the ADNI; the latter was a supplement and identical to the evaluation of the Cog-D group. The MoCA scale applied in cohort 1 was the Chinese MoCA-Basic version, in cohort 2 was the MoCA-Beijing version, and in ADNI was the traditional MoCA version. Continuous measures are presented as mean ± standard deviation. Statistical analysis was conducted using the χ^2^ test for categorical variables and an independent two-sample two-tailed t-test for quantitative variables. *p < 0.05, **p < 0.001. Aβ, amyloid-β; P, positive; N, negative; Cog, cognition; D, deteriorated; M, maintained; MoCA, Montreal Cognitive Assessment; SUVR, standard uptake value ratio; ADNI, Alzheimer’s Disease Neuroimaging Initiative; Ava, available.

In cohort 2, an additional 51 healthy participants were dichotomized according to their future cognitive outcomes. They were interviewed every 10–15 months, and we found the cognition of 24 of these participants deteriorated after an average of 41.2 months [interquartile range (IQR), 24.5–52.7], with 23 progressing to MCI and one to dementia. The others remained healthy after at least three follow-up visits (54.8 months; IQR, 48.9–58.9). As shown in Table 1, there were no differences between the two groups.

Cohort 3 included the 24 converters from cohort 2 as well as 13 from ADNI. Their average score on the MoCA scale dropped from 23.7 ± 2.7 at baseline to 20.5 ± 3.8 at the follow-up time point. The average conversion time of ADNI participants was 62.1 months (IQR, 55.1–66.5), compared to 48.1 months (IQR, 27.8–61.7) across the whole group. The individuals from ADNI were used as an additional supplement, with 12 progressing from cognitively healthy individuals to MCI and one to dementia. Other data are shown in Table 1.

### Radiomics Features Selection

For each participant in the three cohorts, 30,128 features were extracted, including 24,940 features from sMRI, 4,988 from fMRI, and 200 from DTI. To avoid overfitting, these features were screened before being included in the classification models. In the training set, 9,000–11,000 features were retained after the two-sample *t*-tests (*p* < 0.05) and 2,200–2,500 types of uncorrelated features were reserved after the autocorrelation tests. The remaining features were further filtered by three independent selection algorithms. More specifically, we retained the top 50 ranked features using the Fisher score test, 50–70 features using the Lasso method, and the top 50 ranked features after the mRMR test.

Generally, the retained features were consistent in repeated experiments. As shown in Table 2, there were 10 high-frequency features of each composite function disturbance; notably, they were all based on the sMRI modality. For the features selected from the disturbance containing the Fisher score test, the frequency was 420–500 times, mainly originating from the posterior cingulate (left, 3/10; right, 3/10). For the features selected from Lasso, the frequency was 383–468, and no specific regions were identified. Regarding the features selected from mRMR, the frequency was 320–495, also mainly from the posterior cingulate (left, 4/10; right, 2/10).

**TABLE 2:** The high-frequency features selected by cross-validation with different methods.

Two-sample *t*-test, autocorrelation, and Fisher score				Two-sample *t*-test, autocorrelation, and Lasso				Two-sample *t*-test, autocorrelation, and mRMR			
Feature (ID)	Times	Brain region	R	Feature (ID)	Times	Brain region	R	Feature (ID)	Times	Brain region	R
LZHGE (6486)	500	Cingulum_Post_L	1/2	Busyness (26056)	468	Frontal_Mid_Orb_R	2	LZHGE (6486)	495	Cingulum_Post_L	1/2
LZHGE (6529)	500	Cingulum_Post_R	1/2	Homogeneity (24775)	467	Vermis_7	3/2	LZHGE (6529)	488	Cingulum_Post_R	1/2
LZHGE (11474)	500	Cingulum_Post_L	2/3	Variance (**27442**)	430	Parietal_Sup_L	2	LZHGE (**11517**)	486	Cingulum_Post_R	2/3
LZHGE (**11517**)	500	Cingulum_Post_R	2/3	Contrast (14273)	419	Cerebelum_6_R	2/3	ZSN (27076)	445	Occipital_Sup_R	2
Variance (**27442**)	480	Parietal_Sup_L	2	Complexity (9287)	402	Cerebelum_6_R	1/2	LZHGE (11474)	441	Cingulum_Post_L	2/3
LZLGE (24803)	447	Vermis_7	3/2	Coarseness (**6489**)	399	Cingulum_Post_L	1/2	Variance (**27442**)	441	Parietal_Sup_L	2
Strength (18834)	428	Temporal_Inf_R	1	Kurtosis (7485)	397	Parietal_Sup_L	1/2	SZLGE (11471)	420	Cingulum_Post_L	2/3
Coarseness (**6489**)	423	Cingulum_Post_L	1/2	Busyness (26314)	394	Cingulum_Ant_R	2	SZLGE (18179)	398	Temporal_Mild_L	1
ZSN (27076)	423	Occipital_Sup_R	2	LZHGE (**11517**)	391	Cingulum_Post_R	2/3	Coarseness (**6489**)	339	Cingulum_Post_L	1/2
GLN (16497)	420	Cingulum_Post_R	1	Kurtosis (5292)	383	Frontal_Mid_R	1/2	ZSV (28977)	320	Cerebelum_Crus2_R	2

*Under the sample disturbance of five-fold cross-validation, we carried out three different kinds of composite function disturbances separately to screen features in the training dataset and repeated the process 100 times. We calculated the number of occurrences of each retained feature, ranging from 0 to 500, and listed the top 10 most frequently appearing features here; they all originated from the sMRI modality. Three stable high-frequency features were verified, and their identification numbers were 11517, 27442, and 6489. The kurtosis feature belongs to the “global” category; the homogeneity and variance features belong to the “gray-level co-occurrence matrix” category; the GLN, ZSN, LZHGE, SZLGE, LZLGE, and ZSV features belong to the “gray-level size zone matrix” category; and the strength, coarseness, busyness, complexity, and contrast features belong to the “neighborhood gray-tone difference matrix” category. Notably, the variance and contrast features could also originate from the “global” and “gray-level co-occurrence matrix” category, respectively. The “R” represents weights to bandpass sub-bands in wavelet filtering. Lasso, least absolute shrinkage and selection operator; mRMR, max-relevance and min-redundancy; ID, identify number; sMRI, structural magnetic resonance imaging; L, left; R, right; Post, posterior; Sup, superior; Inf, inferior; Mid, middle; Orb, orbital; Ant, anterior; GLN, gray-level nonuniformity; ZSN, zone-size nonuniformity; LZHGE, large zone high-gray-level emphasis; SZLGE, small zone low gray-level emphasis; LZLGE, large zone low-gray-level emphasis; ZSV, zone-size variance.*

Three stable high-frequency features were identified during the process as follows: the large zone high-gray-level emphasis (LZHGE) feature of the right posterior cingulate gyrus on sMRI (ID: 11517; 459 times on average), the variance feature of the left superior parietal gyrus on sMRI (ID: 27442; 450 times on average), and the coarseness feature of the left posterior cingulate gyrus on sMRI (ID: 6489; 387 times on average). They were undisturbed by the combined disturbances and may be of great importance in the preclinical stage of AD. Additionally, among features and the number of occurrences greater than 300, another two were also identified as overlaps: the LZHGE feature of the left posterior cingulate gyrus on sMRI (ID: 6486; 458 times on average) and the zone-size variance feature of right cerebellum-crus2 on sMRI (ID: 28977; 319 times on average).

Other retained features that occurred more than 300 times and the meanings of stable high-frequency features are described in Supplementary Table 2 and Material.

### Classification Performance

We introduced two types of models to determine whether the retained features were compatible for classification analysis. Table 3 presents the classifier performance results in terms of accuracy, sensitivity, and specificity. As shown, the SVM model (radial basis kernel) showed excellent classification efficiency, with an average accuracy of up to 90.2–95.9% (sensitivity, 85.9–92.8%; specificity, 93.7–98.3%) in the validation set and 84.5–88.9% (sensitivity, 79.8–82.9%; specificity, 86.0–96.7%) in the test set. Similar results were obtained in the RF model (Table 3) or the SVM models with the other two kernels (Supplementary Table 3). In contrast, the average accuracy of pure clinical data–based models in diagnosing preclinical AD reached only random-level accuracy at 55.9–56.0% (details are presented in the Supplementary Table 4 and Material).

**TABLE 3:** Classification performance of the SVM model (radial basis kernel) and RF model.

		SVM model (radial basis kernel)			RF model		
Group	Method	ACC	SEN	SPE	ACC	SEN	SPE
Validation dataset	Fisher score	90.23% ± 4.78%	85.91 ± 8.94%	93.71 ± 5.45%	87.07 ± 5.53%	80.29 ± 10.53%	92.46 ± 5.85%
Test dataset	Fisher score	84.48 ± 5.58%	82.87 ± 12.56%	85.98 ± 7.11%	83.19 ± 5.89%	77.80 ± 12.38%	88.19 ± 6.47%
Validation dataset	Lasso	**95.90 ± 3.29%**	**92.82 ± 6.84%**	**98.26 ± 2.80%**	90.81 ± 4.76%	84.58 ± 9.13%	95.74 ± 4.60%
Test dataset	Lasso	**88.94 ± 5.33%**	**80.56 ± 10.71%**	**96.70 ± 3.83%**	83.68 ± 6.85%	74.26 ± 13.86%	92.41 ± 5.78%
Validation dataset	mRMR	93.00 ± 4.19%	89.07 ± 7.11%	96.08 ± 3.98%	90.28 ± 4.97%	84.14 ± 10.03%	95.08 ± 4.96%
Test dataset	mRMR	86.08 ± 5.68%	79.76 ± 12.13%	91.93 ± 5.00%	83.52 ± 6.62%	75.85 ± 13.47%	90.63 ± 6.01%

*Under the sample disturbance of five-fold cross-validation, we carried out three different kinds of composite function disturbances separately to screen features in the training dataset and repeated the process 100 times. The retained features were incorporated into the SVM model and RF model each time, and we then calculated the models’ classification performance in the validation dataset and test dataset separately. The measures are presented as mean ± standard deviation. SVM, support vector machine; RF, random forest; ACC, accuracy; SEN, sensitivity; SPE, specificity; Lasso, least absolute shrinkage and selection operator; mRMR, max-relevance and min-redundancy.*

**FIGURE 2:**
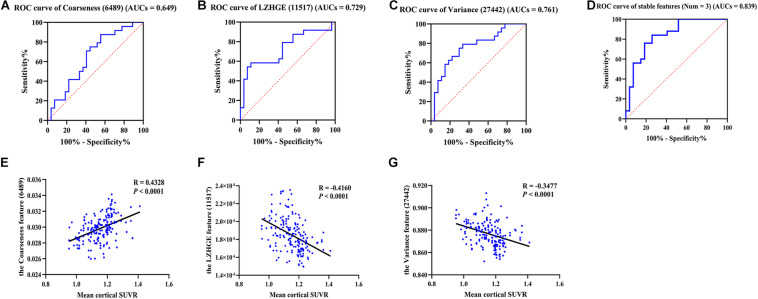
The ROC curves of stable high-frequency features and correlation analysis. **(A–D)** Show the ROC curves of stable high-frequency features in the test dataset; they all have high discriminating power. In detail, the 6489 feature AUCs = 0.649 **(A)**, 11517 feature AUCs = 0.729 **(B)**, and the 27442 feature AUCs = 0.761 **(C)**. The value increased to 0.839 when combined **(D)**. **E–G** show the correlations between the levels of these features and the mean cortical SUVR values in participants of cohort 1. The features were the coarseness feature of the left posterior cingulate gyrus on sMRI (ID: 6489; **A,E**), the LZHGE feature of the right posterior cingulate gyrus on sMRI (ID: 11517; **B,F**) and the variance feature of the left superior parietal gyrus on sMRI (ID: 27442; **C,G**). LZHGE, large zone high-gray-level emphasis; SUVR, standardized uptake value ratio; ROC, receiver operating characteristic; AUCs, areas under curve; sMRI, structural magnetic resonance imaging; Num, number.

We further verified the classification efficiency of stable high-frequency features on the test set and found that their individual AUCs ranged from 0.649 to 0.761, and when we combined them, the predictive ability improved (AUCs = 0.839; Figures 2A–D). In addition, feature 6486 also had a good classification effect (AUCs = 0.739) and improved the AUCs to 0.863 when combined with the three stable features (Supplementary Figure 2). In contrast, the performance of feature 28977 was too poor to create an ROC curve. These results indicate that radiomics analysis is a reliable feature extraction method in the preclinical stage of AD and provides promising imaging biomarkers for identifying cognitively healthy individuals that go on to experience future cognitive decline.

### Correlation Analysis

In order to further understand the association between radiomics features and pathological changes in AD, we performed a correlation analysis between stable high-frequency features and mean cortical SUVR values on amyloid PET and found that they were highly correlated. In detail, feature 6489 levels were positively correlated with SUVR values (*r* = 0.433, *p* < 0.0001, Figure 2E), whereas the feature 11517 and 27442 levels were both inversely correlated with SUVR values (*r* = -0.416, *p* < 0.0001, Figure 2F; *r* = -0.348, *p* < 0.0001, Figure 2G). Similar results were found for feature 6486 (*r* = -0.400, *p* < 0.0001, Supplementary Figure 2). The correlation results did not change after adjusting for age, gender, education, and MoCA score (Supplementary Figure 3). Our findings revealed high correlations between the levels of these features and Aβ deposition, suggesting that radiomics features based on MPMRI may reflect pathological changes in the brain and can be used for the diagnosis of AD.

### Longitudinal Analyses

**FIGURE 3:**
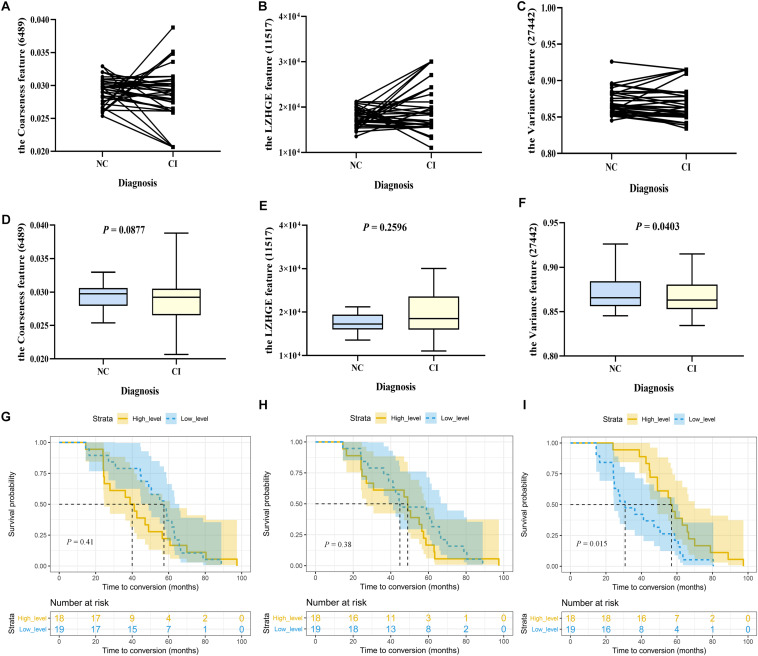
The longitudinal changes and survival analyses of stable high-frequency features. In Study 2, 37 participants were included. Their cognition was normal at baseline and impaired during follow-up, with two progressing to dementia and 35 to mild cognitive impairment. We compared the levels of each stable high-frequency feature between the two time points. **(A–C)** Show the changing trajectory at the individual level; **(D–F)** are at the group level, with paired two-sample *t*-tests (two-tailed, *p* < 0.05). Furthermore, these individuals were stratified into high-level (*n* = 18) and low-level group (*n* = 19) groups by the baseline median level of each stable high-frequency feature. **(G–I)** Show Kaplan–Meier curves demonstrating the cumulative probabilities of conversion of the two groups (shaded area represents the 95% confidence interval); differences are displayed by log-rank tests (*p* < 0.05). Only feature 27,442 was different at the two time points (*p* = 0.0403), and its low-level group had a shorter conversion time than the high-level group (*p* = 0.015). The features were the coarseness feature of the left posterior cingulate gyrus on sMRI (ID: 6489; **A, D, G**), the LZHGE feature of the right posterior cingulate gyrus on sMRI (ID: 11517; **B, E, H**), and the variance feature of the left superior parietal gyrus on sMRI (ID: 27442; **C, F, I**). NC, normal control; CI, cognitive impairment; sMRI, structural magnetic resonance imaging; LZHGE, large zone high-gray-level emphasis.

In this study, 37 participants from cohort 3 were followed up until cognitive impairment was identified. First, we detected the longitudinal changes of each stable high-frequency feature. As shown, features 6,489 and 11,517 did not show isotropic changes in the two cognitive stages at the individual level (Figures 3A,B); correspondingly, there were also no statistical differences between the two paired groups (Figures 3D,E). Similar results were obtained for feature 6486 (Supplementary Figures 4A,B). Although some individuals had a heterogeneous change pattern of feature 27442 (Figure 3C), its levels in the cognitive impairment stage were still lower than those in the cognitively healthy stage (*p* = 0.0403; Figure 3F). Second, we performed survival analyses of these features. In detail, the median baseline levels of features 6489, 11,517, and 27,442 were 0.0297356, 17228.308, and 0.865647, respectively; Figures 3G–I show the probability of cognitive impairment by levels of features > and ≤ these cutoffs. Notably, in the comparison between paired groups, only grouping by feature 27,442 was meaningful (log rank *p* = 0.015). The result of feature 6,486 was also unsatisfactory when grouped by the median level of 48.967 (log-rank *p* = 0.442; Supplementary Figure 4C). These results indicated that the levels of feature 27,442 decreased with cognitive decline, and the deterioration occurred earlier when the baseline level was less than 0.865647. However, considering the limited sample size, the value is for reference only, and it is more accurate to state that the baseline level can affect the conversion time.

## Discussion

Using cross-validations with widely used machine learning techniques, this study demonstrated that radiomics features appear to be robust imaging biomarkers of preclinical AD. The real pathophysiological process of AD is thought to begin several decades before symptom onset and is generally followed by a rigid progress pattern, such as Aβ accumulation-neurofibrillary tangles-neuronal damage; neurons are already damaged to some extent when cognitive impairment begins (Li T. R. et al., 2019; Long and Holtzman, 2019). Radiomics analysis can extract high-dimensional features of MPMRI and may identify imaging patterns in the preclinical stages that cannot be recognized by human readers; however, there is a paucity of published literature assessing the radiomics features of individuals in the preclinical stage of AD and those who go on to develop future cognitive decline. In this ongoing prospective cohort study, we adopted a novel composite method to select features from the training dataset, established classification models, and verified them in the validation and test sets. We found that both models could distinguish whether individuals were in the preclinical stage of AD or whether their cognition will decline in the future, with an accuracy of more than 80%. In addition, three stable high-frequency features were identified, which were independent of perturbations, correlated with Aβ deposition, and classified the test set accurately (AUCs 0.649–0.761). In the independent longitudinal analyses, we further verified that levels of the feature 27,442 (variance feature of the left superior parietal gyrus on sMRI) decreased with cognitive decline and affected individuals’ conversion time. Together, these data showed that radiomics features of MPMRI could be important imaging biomarkers for identifying patients with preclinical AD.

Our previous studies confirmed that cognitively normal individuals at high risk of developing AD already appeared to have altered brain functional networks (fMRI), white matter networks (DTI), or some refined areas (sMRI) (Li et al., 2016; Shu et al., 2018; Yan et al., 2018; Zhao et al., 2019), suggesting that there may be more unmined MPMRI data in the preclinical stage of AD. As expected, the pure clinical data–based classification models were meaningless at this stage, and the traditional volumetric and functional indices were also not sensitive enough (details are presented in the Supplementary Table 5 and Material). Although it is generally believed that radiomics analysis is more sensitive, current studies are still limited to the symptomatic stages of the disease (Alves et al., 2012; Chaddad and Niazi, 2018; Feng F. et al., 2018; Kun et al., 2020; Lee et al., 2020). Chaddad et al. found that the features derived from a single subcortical region produced AUCs up to 80% for identifying AD–dementia in healthy individuals and reached 91.5% when combined with all regions (Chaddad and Niazi, 2018). By using hippocampal features, researchers can distinguish AD–dementia with an accuracy of 86.7 and 70.5% of MCI (Feng F. et al., 2018). Identical conclusions were obtained in a recent large-scale multicenter study where the hippocampal features served as robust biomarkers for clinical identification of AD–dementia/MCI and further predicted whether MCI patients would convert to dementia (Kun et al., 2020). In contrast, the deep learning method can indeed acquire slightly better diagnostic capabilities in the Alzheimer’s continuum (Jo et al., 2019; Yamanakkanavar et al., 2020); however, it is difficult to explain the clinical correlations between these deep features and AD itself, and notably, Li et al. (2017) have proved that the performance in identifying dementia from controls using radiomics is comparable to deep learning (91.4 and 93.9%, respectively). Here, in distinguishing preclinical AD patients or clinical converters, the accuracy of our models reached 81.9–95.9%, even higher than when distinguishing symptomatic patients from controls. We believe that several reasons may account for this. First, compared with extracting features solely on sMRI, we utilized MPMRI. Second, instead of selecting regions based on prior knowledge, we adopted template segmentation and extracted features. Third, among the 30,128 features, we used an innovative selection method to improve robustness. Four, we diagnosed individuals based on Aβ profile and not purely on clinical data, significantly reducing the heterogeneity of participants. Moreover, the use of different types of models further verified the reliability of our findings.

The Aβ deposition associated with neuronal degeneration may have resulted in subtle alterations in MRI signal intensity; therefore, we speculate that radiomics features could reflect changes at the microscopic level during the early pathological stages, which occur before changes at the macroscopic level. In addition to the computer-aided classification, three stable high-frequency features that were not affected by function perturbations (three different algorithms) and sample perturbations (five-fold cross validation and 100 repetitions) were identified during the selection process: the LZHGE feature of the right posterior cingulate gyrus, the variance feature of the left superior parietal gyrus, and the coarseness feature of the left posterior cingulate gyrus (all on the sMRI modality). Importantly, the earliest accumulation of Aβ deposition is also in the superior parietal gyrus and posterior cingulate (Long and Holtzman, 2019). More specifically, in symptomatic AD patients, previous autopsy findings and amyloid-PET studies have suggested that the parietal lobe and posterior cingulum are vulnerable to Aβ invasion during the early stages of AD (Thal et al., 2002; Cho et al., 2018). In cognitively normal individuals, the annual increase in Aβ also localizes to these two regions (Sojkova et al., 2011). From other perspectives, Aβ deposition is associated particularly with cortical atrophy of the superior parietal gyrus (Becker et al., 2011; Weston et al., 2016) and the rate-limiting enzyme of Aβ production is also significantly elevated in this area (Coulson et al., 2010). These developments prove the accuracy of the identified anatomical locations and support our findings that these features were correlated with SUVR values and played a good role in predicting future cognitive decline (AUCs 0.649, 0.729, and 0.761, respectively; 0.839 when combined) and thus probably represent the imaging biomarkers of preclinical AD. Interestingly, we found that retained features only came from the sMRI modality, which is probably in part due to the relatively small number of fMRI and DTI features utilized in our study. Additionally, a recent study concluded that DTI parameters are not useful for the identification of preclinical AD (Teipel et al., 2019). To the best of our knowledge, this is the first time that texture analysis of fMRI has been applied to the field of AD (Hassan et al., 2016). Uncertainty still exists, and the significance of DTI and fMRI radiomics features cannot be completely ruled out in this exploratory study.

In the longitudinal analyses, we found that the variance feature of the left superior parietal gyrus on sMRI decreased with cognitive impairment, suggesting that it may be of great importance in the whole cognitive continuum and not just in the preclinical stage. This feature is extracted from the gray-level co-occurrence matrix category and is an indicator of dispersion of the unit values around the mean. With cognitive decline, the cortical accumulation of Aβ will increase continuously to a certain extent (Long and Holtzman, 2019) and may result in alterations in signal intensity, with subtle changes captured by the radiomics analysis of sMRI. Next, we conducted survival analyses to compare the conversion time between groups within cohort 3. The median value was chosen subjectively for grouping; coincidently, we found that the variance feature can affect the conversion time, further suggesting its predictive effects on clinical outcome.

Our study had some limitations. First, the small sample number limited the statistical power of our data. We tried to overcome this issue by enrolling participants from other subcenters and the ADNI, but the requirement of amyloid PET, long-term follow-up, or MPMRI data greatly limited the quantity of potential participants. Moreover, the performance of our models may differ when using different imaging protocols. Second, considering there is no standard definition of “unstable preclinical AD”, we referred to the 36 months of “unstable MCI” and required the nonconverters to remain cognitively stable for at least three follow-up visits. The average conversion time of converters was 41.2 months, which needs to be verified. Third, other anatomical regions, such as the anterior cingulate, are also susceptible to Aβ attack (Thal et al., 2002; Cho et al., 2018). However, we did not find any stable features in these regions; some high-frequency features came from regions that are not or are weakly related to AD, such as the cerebellum, and it is difficult to associate these regions with clinical significance. Fourth, in cohort 3, most of the patients were limited to the MCI stage and few to the dementia stage at the follow-up time point; thus, it is not clear whether features were related to the degree of cognitive deterioration. Fifth, the positive result of Study two was not significant (*p* = 0.0403), and the feature levels of some individuals increased disparately, which was probably due to the heterogeneity of MCI and the relatively older age of the ADNI participants. Sixth, age may cause differences in our results because of its impact on Aβ and atrophy. Seventh, the positive rate of amyloid PET (42.6%) was higher than that reported in previous studies (10–30% mostly) (Chételat et al., 2013), partly because of the exclusion of some negative individuals (Supplementary Figure 1A) and the existence of individuals with subjective cognitive decline, itself a high-risk state for developing AD (Jessen et al., 2020). This bias may increase uncertainty. Eighth, different guidelines have inconsistent definitions of preclinical AD (Dubois et al., 2007, 2010, 2014; Sperling et al., 2011a; Jack et al., 2018). The latest NIA-AA 2018 definition requires additional evidence of tau deposition in patients with preclinical AD (Jack et al., 2018), but the tau status of participants was not clear in our study. Considering these limitations, multicenter collaboration to include more participants is needed in the future.

In conclusion, radiomics analysis of MPMRI is expected to become a new evaluation method for Aβ deposition and future cognitive decline in cognitively healthy individuals, which would be of great importance in diagnosing preclinical AD and targeting ultra-early secondary prevention clinical trials. Additionally, we have proposed a novel feature extraction paradigm and preservation method for feature subsets, solving the problem of instability and nonrepeatability for future studies.

## Data Availability Statement

The datasets used and/or analysed during the current study are available from the corresponding authors on reasonable request.

## Ethics Statement

The study was approved by the Medical Ethics Committee of Xuanwu Hospital of Capital Medical University and was carried out in accordance with the Declaration of Helsinki. We confirm that we have read the Journal’s position on issues involved in ethical publication and affirm that this report is consistent with those guidelines. All subjects gave written informed consents and written consent to permit publication of clinical details.

## Author Contributions

YH and T-RL provided the data. J-HJ designed the study. T-RL and YW assembled and analyzed the data, consulted literatures, and drafted the manuscript. J-JJ drawn the Figure 1. HL and C-LH polished the manuscript. YH and J-HJ critically revised the manuscript for important intellectual content. All authors read and approved the final manuscript.

## Conflict of Interest

The authors declare that the research was conducted in the absence of any commercial or financial relationships that could be construed as a potential conflict of interest.

## Footnotes

1 www.loni.ucla.edu/ADNI

2 https://ida.loni.usc.edu/login.jsp

3 https://people.cas.sc.edu/rorden/mricron/dcm2nii.html

4 http://www.rfmri.org/DPARSF

5 https://www.fil.ion.ucl.ac.uk/spm/software/spm12

6 https://github.com/mvallieres/radiomics
