# Supplementary figures and images for: Radiomics Analysis of Magnetic Resonance Imaging Facilitates the Identification of Preclinical Alzheimer’s Disease: An Exploratory Study
Source: Front Cell Dev Biol. 2020 Dec 3;8:605734. doi: 10.3389/fcell.2020.605734 (PMC7744815; doi:10.3389/fcell.2020.605734)

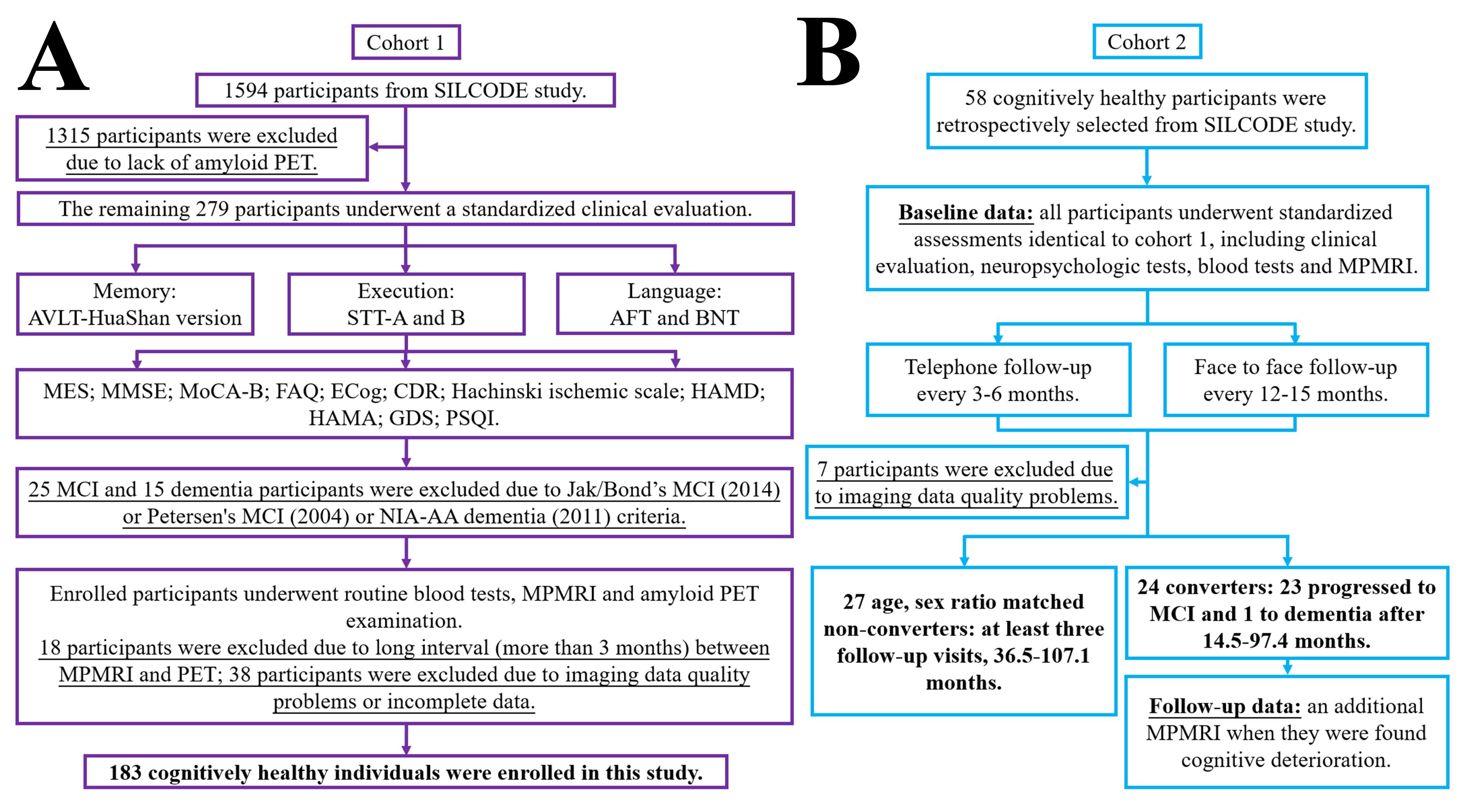

Supplement: Supplementary file 1 [file Image_1.TIF]

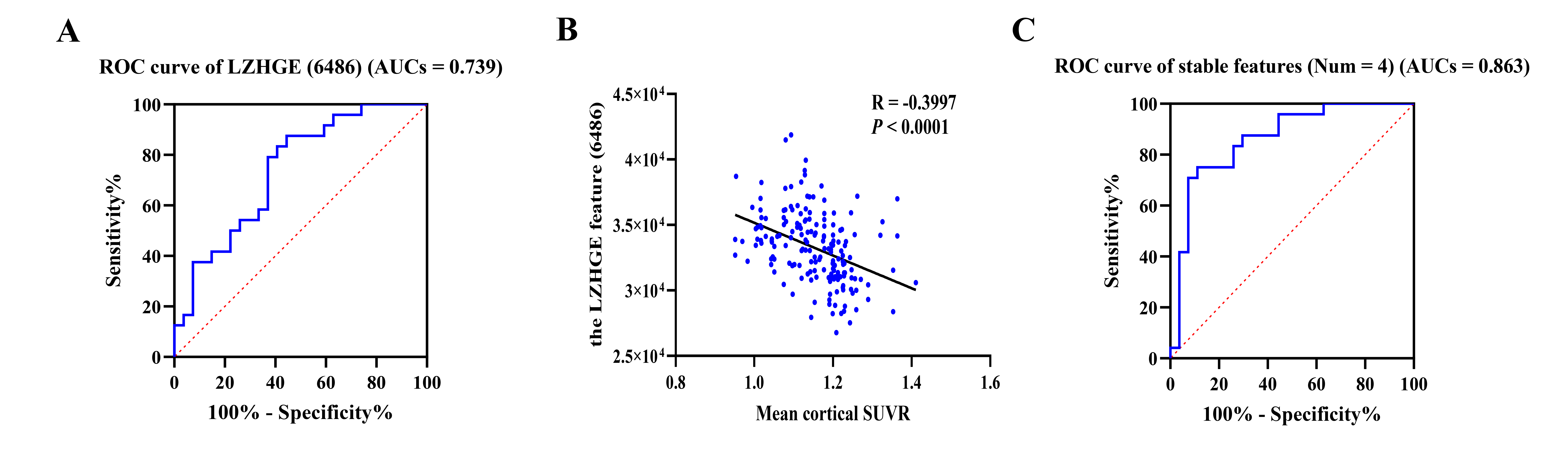

Supplement: Supplementary file 2 [file Image_2.TIF]

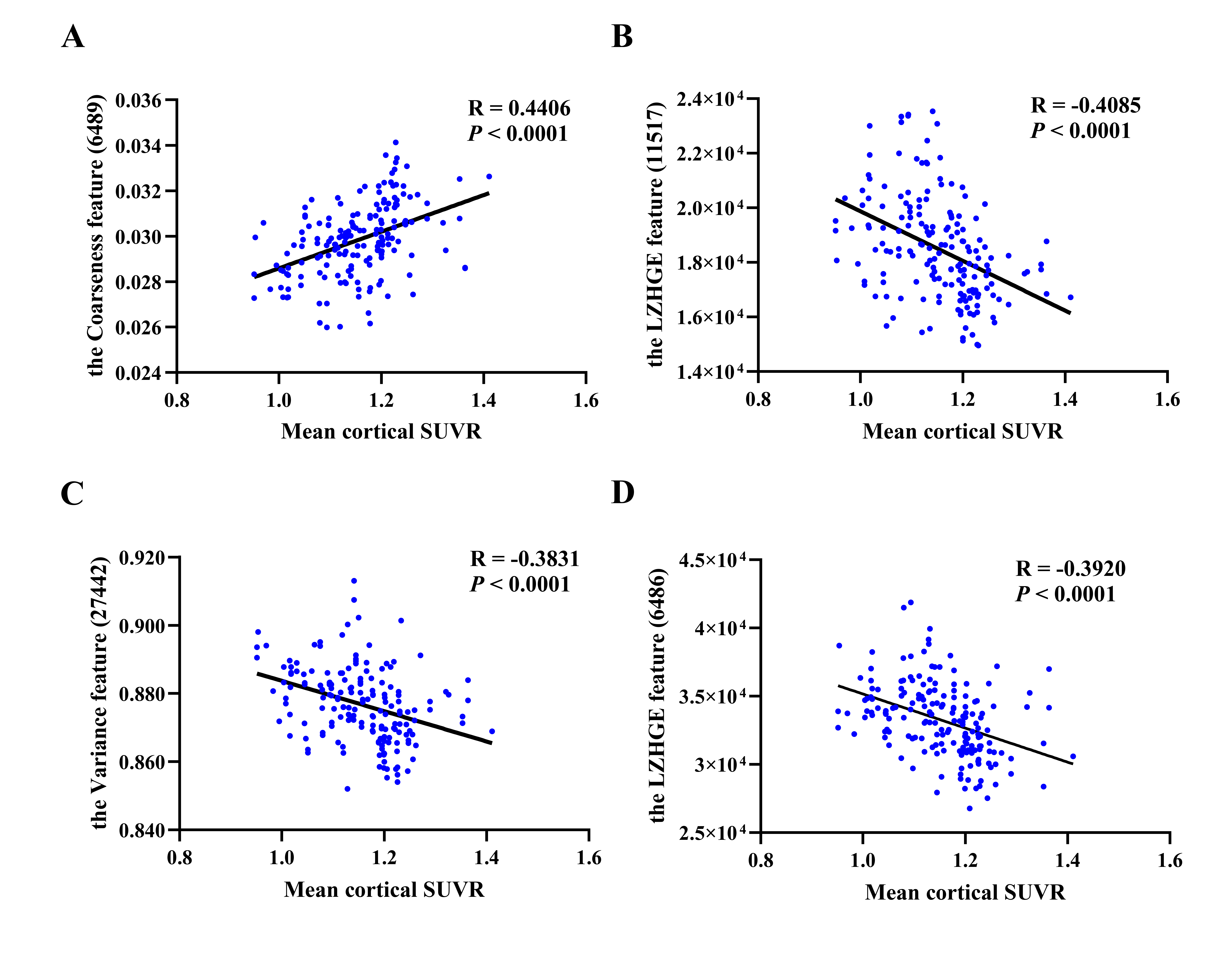

Supplement: Supplementary file 3 [file Image_3.TIF]

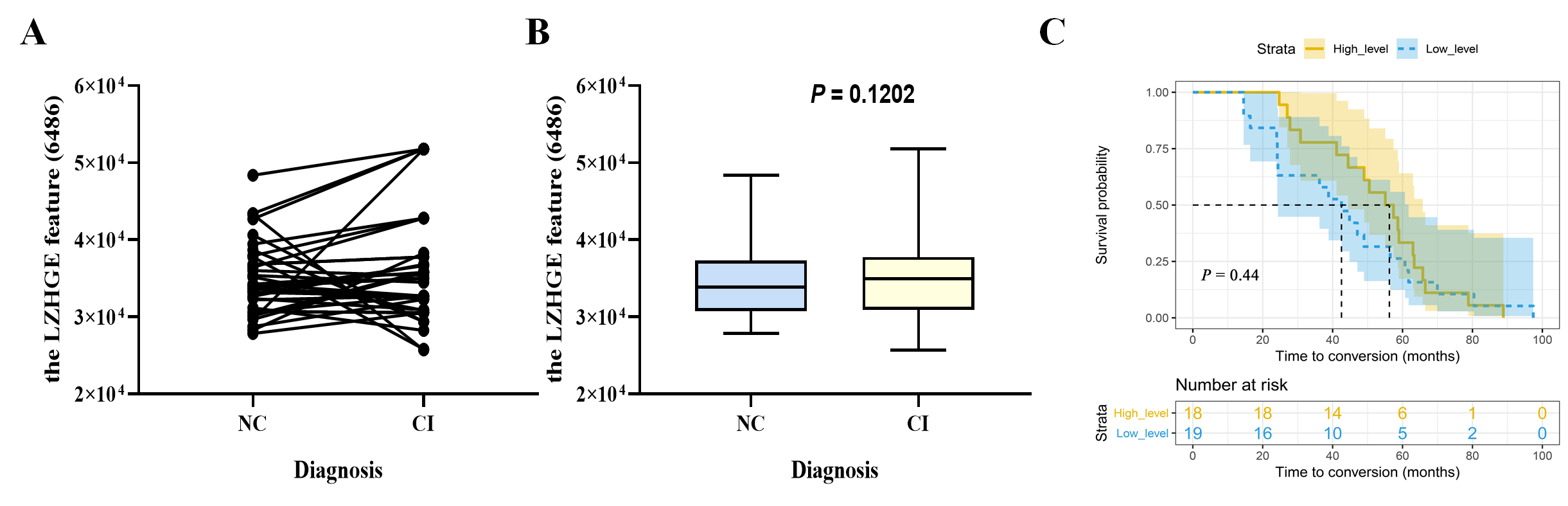

Supplement: Supplementary file 4 [file Image_4.TIF]
