# Supplementary material for: Radiomics Analysis of Magnetic Resonance Imaging Facilitates the Identification of Preclinical Alzheimer’s Disease: An Exploratory Study
Source: Front Cell Dev Biol. 2020 Dec 3;8:605734. doi: 10.3389/fcell.2020.605734 (PMC7744815; doi:10.3389/fcell.2020.605734)
Supplement: Supplementary file 5 [file Data_Sheet_1.docx]

**Supplementary Material**

**1. Participants**

The Sino Longitudinal Study on Cognitive Decline (SILCODE) (*ClinicalTrials.gov* identifier: NCT03370744) is a research continuation of previous studies (*ClinicalTrials.gov* identifier: NCT02353884 and NCT02225964) (Li et al., 2019). All participants were rigorously evaluated in a standardized protocol, including a medical history interview, neurologic examination, and a battery of neuropsychologic tests. The tests included auditory verbal learning test (AVLT)-long delayed memory and recognition for memory domain assessment, animal fluency test (AFT) and 30-item Boston naming test (BNT) for language domain assessment, shape trails test (STT)-part A and B for speed and executive domain assessment, memory and execution screening (MES), mini-mental state examination (MMSE) and Montreal Cognitive Assessment-basic (MoCA-B, or Beijing Version) for global cognition assessment, functional activities questionnaire (FAQ) for daily life ability assessment, and everyday cognition scale (ECog), clinical dementia rating (CDR), Hamilton depression scale (HAMD), Hamilton anxiety scale (HAMA), geriatric depression scale (GDS), Pittsburgh sleep quality index (PSQI) for neuropsychiatric assessment and others. These tests were performed by experienced neurologists of the same research group, and the diagnosis was checked by two experienced neurologists (Ying Han and Tao-Ran Li).

The entry criteria of healthy individuals referred to our previous references (Chen et al., 2019; Li et al., 2019), briefly as follows: 60-79 years old, right-handed Mandarin-speaking; exclusion of dementia or mild cognitive impairment (MCI); neuropsychological tests scores were in normal range. Notably, the optimal cutoffs were corrected by gender, age and years of education, and suitable for the Chinese (Lu et al., 2011; Chen et al., 2016; Li et al., 2019), and part of the subjects in cohort 1 were individuals with subjective cognitive decline from the communities, and in accordance with the research framework of the National Institute on Aging-Alzheimer’s Association (NIA-AA) (Jack Jr et al., 2018), study participants with subjective cognitive decline were analyzed together with cognitively healthy participants (and combined in the cohort 1). Participants were excluded if they had history of stroke, severe anemia, syphilis infection, or other conditions (Li et al., 2019). The diagnosis of dementia had been established according to the NIA-AA workgroups (McKhann et al., 2011), and of MCI was based on Petersen’s criteria (before 2016) (Petersen, 2004) or a neuropsychological method (after 2016) (Bondi et al., 2014).

For the 51 participants of cohort 2, in addition to the baseline examinations, they were followed face-to-face interview every 10-15 months, and performed neuropsychologic tests at each time.

The Supplementary Figure 1 showed the recruitment process of participants of our two cohorts.

In order to further expand the sample size, we tried to get participants from the Alzheimer's Disease Neuroimaging Initiative (ADNI, [www.loni.ucla.edu/ADNI](http://www.loni.ucla.edu/ADNI)). One hundred and three individuals meet the following requirements: the cognition was normal at baseline and deteriorated to MCI or dementia during the follow-up period; we can acquire the data of multi-parametric magnetic resonance imaging (MPMRI) and neuropsychological examinations at both baseline and follow-up time points. However, after excluding the imaging data that cannot be decompressed, preprocessed and (or) features extracted, only 13 individuals remained, and being included for longitudinal researches.

**2. Imaging acquisition protocols**

The MPMRI data were acquired using a 3.0-T MRI scanner (Siemens) before the year of 2016 or an integrated simultaneous 3.0-T time-of-flight PET/MR (GE) after that time point.

Three-dimensional T1-weighted magnetization-prepared rapid gradient-echo sagittal images were obtained by using the following sequence (Siemens/GE): field of view (FOV) = 256×256 mm^2^/256×256 mm^2^, matrix = 256×256/256×256, slice thickness = 1 mm/1 mm, gap = 0/0, slice number = 176/192, repetition time (TR) = 1900 ms/6.9 ms, echo time (TE) = 2.2 ms/2.98 ms, inversion time (TI) = 900 ms/450 ms, flip angle = 9^°^/12^°^, voxel size = 1×1×1 mm^3^/1×1×1 mm^3^.

For the images of resting state-functional magnetic resonance imaging (rs-fMRI), a single-shot gradient-echo echo planar imaging (EPI) sequence was used with the following parameters (Siemens/GE): scan duration = 8 min/8 min, FOV = 240×240 mm^2^/224×224 mm^2^, data matrix = 64×64/64×64, slice thickness = 4.0 mm/3.5 mm, gap = 1.0 mm/0.7 mm, slice number = 28/28, slice order = interleaved, TR = 2000 ms/2000 ms, TE = 40 ms/30 ms, flip angle = 90^°^/90^°^, voxel size = 3.75×3.75×4.0 mm^3^/3.5×3.5×4.0 mm^3^.

For the diffusion tensor imaging (DTI), the data were obtained with a single-shot spin-echo diffusion-weighted EPI sequence as the following parameters (Siemens/GE): FOV = 256×232 mm^2^/224×224 mm^2^, data matrix = 128×116/112×112, slice thickness = 2 mm/2 mm, gap = 0/0, slice number = 60/75, slice order = interleaved, TR = 11000 ms/16500 ms, TE = 98 ms/95.6 ms, 30/30 gradient directions and 1/10 b0 images (b = 1000 s/mm^2^/1000 s/mm^2^), voxel size = 2×2×2 mm^3^/2×2×2 mm^3^.

The amyloid-positron emission tomography (PET) images were acquired 40 min after intravenous injection of Florbetapir F-18 (AV45; 7-10 mCi). The participants received a 35 min dynamic scan and the data were recorded by using a TOF-OSEM algorithm (time-of-flight ordered subset expectation maximisation) with the following parameters: eight iterations, 32 subsets matrix = 192×192, FOV = 350×350, half-width height = 3.

**3. Regions of interest**

Based on the AAL (Anatomical Automatic Labeling) template, we considered the 116 brain regions (90 of cerebrum and 26 of cerebellum) as regions of interest (ROIs).

**4. Feature extraction**

The feature extraction of sMRI and rs-fMRI was performed on the Texture Toolbox of radiomics tools developed by *Vallieres et al.* (<https://github.com/mvallieres/radiomics>), based on MATLAB (Vallières et al., 2015). The extraction was operated on each ROI separately, including matrix-based texture analysis and wavelet analysis. For the sMRI data, the total steps included wavelet band-pass filtering, isotropic resampling, Lloyd-Max quantization, and features calculation. First, we carried out wavelet band-pass filtering by assigning different weights to bandpass sub-bands (LHL, LHH, LLH, HLL, HHL, and HLH) of the volume of interest in the comparison with low- and high-frequency sub-bands (LLL and HHH) in the wavelet domain; the ratio of weight was defined as R, and the values of R were 1/2, 2/3, 1 (no wavelet filtering), 3/2, and 2, respectively; the global features were extracted before the following steps. Second, we performed isotropic resampling with an initial in-plane resolution for each image. Third, we adopted the Lloyd-Max quantization algorithm to normalize each image to 256-gray-level. After these steps, we can obtain three histogram-based textures and four types of texture matrices by quantizing these images, specifically as follows: Variance, Skewness, Kurtosis, gray-level co-occurrence matrix (GLCM), gray-level run-length matrix (GLRLM), gray-level size zone matrix (GLSZM), and neighborhood gray-tone difference matrix (NGTDM). Based on these texture matrices, 43 texture features and 172 wavelet features were acquired. The features extraction of rs-fMRI data was consistent with sMRI except that there was no wavelet filtering. Therefore, we only obtained 43 texture features from each ROI of the rs-fMRI images. The 43 texture features including 3 histogram-based textures, 9 texture features from the GLCM, 13 texture features from the GLRLM, 13 texture features from the GLSZM, and 5 texture features from the NGTDM. The details were listed in the Supplementary Table 1.

For DTI, we calculated 50 white matter tracts, which defined by the International Consortium for Brain Mapping DTI-81 (ICBM DTI-81) atlas, and defined the fractional anisotropy, mean diffusivity, axial diffusivity, and radial diffusivity as radiomics features, thus 200 DTI measures were acquired for each participant.

**5. Features dimensionality reduction and selection**

In the process of dimensionality reduction and selection of the acquired features, three steps were performed as follows. First, a two-sample *t*-test was used to retain features with statistical differences (*p*<0.05). Second, we performed autocorrelation tests to reduce the redundancies between high-dimensional features. Based on the pair-wise correlations, we defined and calculated the average absolute correlation value of each feature by the following formula:

$C= \frac{1}{n}\sum_{j} C_{i,j}$ (1)

If the Ci,j value in the pair-wise correlation was greater than 0.8, we thought the paired features had a high correlation and abandoned the feature with a higher average absolute correlation value (Xia et al., 2018). Third, we used three independent feature selection algorithms (Fisher score, Least absolute shrinkage and selection operator [Lasso] and Max-Relevance and Min-Redundancy [mRMR]) to further filter the remaining features; the purpose of the parallel experiments was to verify the stability of retained features and reflect the independence of algorithms. The Fisher score algorithm is a filter-based and supervised feature selection method, could select features according to their scores calculated by Fisher’s criterion independently, in this study, the top-50 ranked features were selected for subsequent analyses. For the Lasso algorithm, we retained non-zero coefficient features ranged from 50-70. Last, we used the mRMR algorithm to maximize the correlation between features and categorical variables as well as minimize the correlation between features, and retained the first 50 features.

It should be noted that the three algorithms belong to two different kinds of feature selection methods, more specifically, the Fisher score algorithm and mRMR algorithm are both filter-based method, selecting features based on the correlation between independent variables and target variables, and scoring each feature according to the divergence or correlation; while the Lasso algorithm belongs to the ‘embedded’ method, it trains the model based on the algorithm and gets the weight coefficient of each feature, then selects features according to the coefficient (Chandrashekar and Sahin, 2014). Therefore, the selected features are relatively reliable and repeatable at least at the technical level. In order to ensure the consistency of classification objective function and favorable classification performance, the ‘Wrapper’ method, such as the support vector machine (SVM)-recursive feature elimination (RFE) algorithm, was not selected, because it is based on the objective function to determine whether to include features, may result in overfitting or other problems in the SVM classifier. The ridge regression is another ‘embedded’ method, compared with Lasso regression, which adds L2 regularization on the basis of standard linear regression, it adds L1 regularization; both the two methods can be used to solve the overfitting problem of standard linear regression, but the Lasso is more likely to make the weight zero, while the ridge regression is more likely to make the weight close to zero, which means that it’s easier to obtain ‘sparse’ solutions in the Lasso, and the parameters in the loss function have fewer non-zero vectors. These above characteristics determine that Lasso is more stable and more suitable for features selection, therefore, we chose it instead of ridge regression.

**6. Classification models establishment**

Before making classifications, we normalized the retained features by using Min-Max normalization method. Then, we conducted classification experiments with the SVM classifier and random forest (RF) classifier.

For the establishment of SVM, all the parameter settings were default, and three kernels (linear, radial basis, sigmoid) have been used to detect the generalization ability of features and classification reliability. The SVM classifier version was LIBSVM 2.9.1 (<https://www.csie.ntu.edu.tw/~cjlin/libsvm/>). For RF, all the parameters were set by default except 20 of the number of trees. We calculated the mean (± standard deviation) accuracy, sensitivity, and specificity of the 100 repetitions as the final result.

All the classification experiments were implemented in MATLAB.

**7. Typical retained features**

The Supplementary Table 2 is a duplication and continuation of Table 2 of the original paper, described the retained features with the number of occurrences more than 300 times. The meanings of stable high-frequency features are as follows: the Large zone high-gray-level emphasis (LZHGE) feature extracted from the GLSZM counts in 3 dimensions the number of zones of voxels presenting with the same grey level discretization, and it emphasizes zone counts in the lower right quadrant of the GLSZM, where large zone sizes and high grey levels are located (Aide et al., 2020); the Variance feature extracted from the GLCM category is an indicator of dispersion of the unit values around the mean (Pantic et al., 2020); the Coarseness feature extracted from the NGTDM has been likened to granularity within an image, that is, coarseness is higher in images of larger granularity and lower in those with a smaller granularity (Cheng et al., 2013).

**8. Classification performance of the SVM models (supplement)**

As shown in the Supplementary Table 3, the classification performance of Linear kernel-based and Sigmoid kernel-based SVM models was similar to that of Radial basis kernel-based model in the original paper. Their average accuracy fluctuated between 89.6–95.9% in the validation set, and 81.9–89.1% in the test set.

**9. Other retained features**

In addition to the three stable high-frequency features, another two features, the Large zone high-gray-level emphasis feature of the left posterior cingulate gyrus on sMRI (ID: 6486) and the Zone-size variance feature of the right cerebellum-crus2 on sMRI (ID: 28977), were both appeared in each selection method with the number of occurrences more than 300 times (Supplementary Table 2). Then, we did further classification experiments on the test data and found the feature 6486 also played a good classification effect (areas under curve [AUCs] = 0.739) (Supplementary Figure 2A), similar to the stable high-frequency features (AUCs = 0.649–0.761); while the performance of feature 28977 was too bad to draw a ROC curve. Compared with the predictive ability of the combined three stable high-frequency features (AUCs = 0.839), the AUCs improved to 0.863 when additionally combined the feature 6486 (Supplementary Figure 2C).

Moreover, the feature 6486 levels were also inversely correlated with the standardized uptake value ratio values (r = -0.400, *p*<0.0001) (Supplementary Figure 2B), and the results did not change after adjusting for variables (Supplementary Figure 3). We didn’t make correlation analysis of the feature 28977 because of its poor classification results and the incomprehensibility of the cerebellum region.

**10. Clinical models**

In order to know whether the preclinical Alzheimer’s disease patients could be identified by clinical information, we established classification models in cohort 1 again by using clinical data purely. The clinical information included demographic data, APOE status and neuropsychological tests (AVLT, AFT, BNT, STT-A, STT-B, MES, MMSE, MoCA, FAQ, HAMD, HAMA, GDS, CDR, ECog and PSQI) scores, which were considered as features. Before being incorporated into models, these features were adjusted to control the impacts of age, gender, and education, and normalized to the range of 0-1. Identically, we performed a five-fold cross-validation on the dataset, the data was randomly divided into a training set (80%) and a validation set (20%) at each time. Then, we established the SVM and RF models respectively based on the training set, and made classifications of the validation set. The above steps were also repeated 100 times, therefore we can acquire the final mean value of accuracy, sensitivity, and specificity, as shown in the Supplementary Table 4. Considering the poor classification accuracy in the two sets, we did not conduct additional experiments in the test set.

**11. Traditional imaging indices**

In order to verify whether the traditional conventional indicators have changed in the preclinical stage, and whether the radiomics analysis is more sensitive than these indicators, the volumes of hippocampi, basal forebrain, entorhinal cortex and whole brain, and the amplitude of low frequency fluctuation (ALFF), fractional ALFF (fALFF) and regional homogeneity (ReHo) of the default mode network (DMN) of participants in our two cohorts were additionally calculated.

The calculation process of ALFF, fALFF and ReHo referred to previous references (Huang et al., 2017; Li et al., 2019). For the analysis of ALFF and fALFF, the time series of each voxel was transformed into the frequency domain using a fast Fourier transform, and the square root of power spectrum was calculated and averaged across 0.01–0.1 Hz, finally, this averaged square root was taken as the ALFF value for this voxel. The fALFF value was the ratio of the power spectrum of low-frequency (0.01–0.1Hz) to that of the entire frequency range. For the ReHo analysis, Kendall’s coefficient of concordance (KCC) was computed on the ranked time series of a given voxel with its 27 nearest neighbors, and the resultant KCC was taken as the ReHo value. The generated ALFF, fALFF and ReHo images were used for statistical analysis. Afterwards, we calculated the average values based on the DMN area. We used the software CAT12 (SPM12, MATLAB) to process the sMRI images and obtain the volumes of hippocampi, basal forebrain, entorhinal cortex, and whole brain.

As shown in Supplementary Table 5, these imaging data are summarized as mean ± standard deviation. The between-group comparisons were performed by using two-sample *t*-tests (two-tailed). A *p*<0.05 was considered significant. In cohort 1, there were no statistical differences of these traditional conventional indicators; in cohort 2, at baseline, individuals with future cognitive decline tended to have a higher ReHo of DMN (*p*=0.012), and decreased volumes of hippocampus (*p*<0.0001), basal forebrain (*p*=0.022) and entorhinal cortex (*p*=0.023) when compared with these ‘non-converters’. Although the regional ReHo and volumes have altered in these individuals, however, it was probably due to a state of impending disease progression (Perrotin et al., 2015; Kang et al., 2017; Perrotin et al., 2017; Hong et al., 2019). These results indicated that traditional imaging indicators are not sensitive enough in the ultra-early period of AD.

| **Supplementary Table 1. Details of the radiomics features** | | |
| --- | --- | --- |
| **Feature category** | **Feature nomenclature** | **Reference** |
| **Global** | Variance; Skewness; Kurtosis | (Rizzo et al., 2018) |
| **GLCM** | Energy; Contrast; Correlation; Homogeneity; Variance; Sum Average; Entropy; Auto Correlation; Dissimilarity | (HARALICK, 1973) |
| **GLRLM** | Short-run emphasis (SRE); Long-run emphasis (LRE); Gray-level nonuniformity (GLN); Run-length nonuniformity (RLN); Run percentage (RP) | (Mary et al., 1975) |
|  | Low gray-level run emphasis (LGRE); High gray-level run emphasis (HGRE) | (Chu et al., 1990) |
|  | Short-run low gray-level emphasis (SRLGE); Short-run high gray-level emphasis (SRHGE); Long-run low gray-level emphasis (LRLGE); Long-run high gray-level emphasis (LRHGE) | (Dasarathy and Holder, 1991) |
|  | Gray-level variance (GLV); Run-length variance (RLV) | (Thibault et al., 2009) |
| **GLSZM** | Small zone emphasis (SZE); Large zone emphasis (LZE); Gray-level nonuniformity (GLN); Zone-size nonuniformity (ZSN); Zone percentage (ZP) | (Mary et al., 1975; Thibault et al., 2009) |
|  | Low gray-level zone emphasis (LGZE); High gray-level zone emphasis (HGZE) | (Chu et al., 1990; Thibault et al., 2009) |
|  | Small zone low gray-level emphasis (SZLGE); Small zone high gray-level emphasis (SZHGE); Large zone low-gray-level emphasis (LZLGE); Large zone high-gray-level emphasis (LZHGE) | (Dasarathy and Holder, 1991; Thibault et al., 2009) |
|  | Gray-level variance (GLV); Zone-size variance (ZSV) | (Thibault et al., 2009) |
| **NGTDM** | Coarseness; Contrast; Busyness; Complexity; Strength | (Amadasun and King, 1989) |

Supplementary Table 1. Details of the radiomics features

Abbreviations: GLCM, gray-level co-occurrence matrix; GLRLM, gray-level run-length matrix; GLSZM, gray-level size zone matrix; NGTDM, neighborhood gray-tone difference matrix.

| **Supplementary Table 2.** **The relatively high-frequency features selected by cross-validation with different methods** | | | | | | | | | | | | | |
| --- | --- | --- | --- | --- | --- | --- | --- | --- | --- | --- | --- | --- | --- |
| **Two-sample *t*-test, autocorrelation, and Fisher score** | | | |  | **Two-sample *t*-test, autocorrelation, and Lasso** | | | |  | **Two-sample *t*-test, autocorrelation, and mRMR** | | | |
| **Feature (ID)** | **Times** | **Brain region** | **R** |  | **Feature (ID)** | **Times** | **Brain region** | **R** |  | **Feature (ID)** | **Times** | **Brain region** | **R** |
| LZHGE (**6486**) | 500 | Cingulum_Post_L | 1/2 |  | Busyness (26056) | 468 | Frontal_Mid_Orb_R | 2 |  | LZHGE (**6486**) | 495 | Cingulum_Post_L | 1/2 |
| LZHGE (6529) | 500 | Cingulum_Post_R | 1/2 |  | Homogeneity (24775) | 467 | Vermis_7 | 3/2 |  | LZHGE (**6529**) | 488 | Cingulum_Post_R | 1/2 |
| LZHGE (**11474**) | 500 | Cingulum_Post_L | 2/3 |  | Variance (**27442**) | 430 | Parietal_Sup_L | 2 |  | LZHGE (**11517**) | 486 | Cingulum_Post_R | 2/3 |
| LZHGE (11517) | 500 | Cingulum_Post_R | 2/3 |  | Contrast (14273) | 419 | Cerebelum_6_R | 2/3 |  | ZSN (27076) | 445 | Occipital_Sup_R | 2 |
| Variance (**27442**) | 480 | Parietal_Sup_L | 2 |  | Complexity (9287) | 402 | Cerebelum_6_R | 1/2 |  | LZHGE (11474) | 441 | Cingulum_Post_L | 2/3 |
| LZLGE (24803) | 447 | Vermis_7 | 3/2 |  | Coarseness (**6489**) | 399 | Cingulum_Post_L | 1/2 |  | Variance (**27442**) | 441 | Parietal_Sup_L | 2 |
| Strength (18834) | 428 | Temporal_Inf_R | 1 |  | Kurtosis (7485) | 397 | Parietal_Sup_L | 1/2 |  | SZLGE (11471) | 420 | Cingulum_Post_L | 2/3 |
| Coarseness (**6489**) | 423 | Cingulum_Post_L | 1/2 |  | Busyness (26314) | 394 | Cingulum_Ant_R | 2 |  | SZLGE (18179) | 398 | Temporal_Mild_L | 1 |
| ZSN (27076) | 423 | Occipital_Sup_R | 2 |  | LZHGE (**11517**) | 391 | Cingulum_Post_R | 2/3 |  | Coarseness (6489) | 339 | Cingulum_Post_L | 1/2 |
| GLN (16497) | 420 | Cingulum_Post_R | 1 |  | Kurtosis (5292) | 383 | Frontal_Mid_R | 1/2 |  | ZSV (**28977**) | 320 | Cerebelum_Crus2_R | 2 |
| Coarseness (26441) | 413 | Cingulum_Post_L | 2 |  | LGZE (19854) | 381 | Vermis_8 | 3/2 |  |  |  |  |  |
| LZHGE (21493) | 392 | Cingulum_Post_R | 3/2 |  | LZHGE (**6486**) | 380 | Cingulum_Post_L | 2/3 |  |  |  |  |  |
| LZHGE (19085) | 383 | Cerebelum_3_R | 3/2 |  | SZLGE (11471) | 375 | Cingulum_Post_L | 1 |  |  |  |  |  |
| Kurtosis (5292) | 365 | Frontal_Mid_R | 2/3 |  | GLV (9110) | 369 | Cerebelum_3_R | 2/3 |  |  |  |  |  |
| ZSV (13282) | 360 | Thalamus_L | 1 |  | Strength (9589) | 356 | Cerebelum_10_L | 2/3 |  |  |  |  |  |
| Busyness (26056) | 329 | Frontal_Mid_Orb_R | 2 |  | Complexity (24681) | 345 | Vermis_3 | 2 |  |  |  |  |  |
| GLV (21494) | 320 | Cerebelum_6_R | 2 |  | Contrast (16982) | 335 | Lingual_L | 3/2 |  |  |  |  |  |
| ZSV (**28977**) | 320 | Cerebelum_Crus2R | 2 |  | Coarseness (8381) | 328 | Heschl_L | 1/2 |  |  |  |  |  |
| Contrast (14273) | 319 | Cerebelum_6_R | 2/3 |  | ZSV (**28977**) | 318 | Cerebelum_Crus2R | 2 |  |  |  |  |  |
| SZHGE (11515) | 314 | Cingulum_Post_R | 2/3 |  | SZHGE (18180) | 310 | Pallidum_L | 1 |  |  |  |  |  |
|  |  |  |  |  | HGZE (5406) | 303 | Frontal_Mid_Orb_R | 1/2 |  |  |  |  |  |

Supplementary Table 2. The relatively high-frequency features selected by cross-validation with different methods

Under the sample disturbance of five-fold cross-validation, we carried out three different kinds of composite function disturbances separately to screen features in the training dataset and repeated the process 100 times. We calculated the number of occurrences of each retained feature, ranging from 0 to 500, and listed the top ten most frequently appearing features here; they all originated from the sMRI modality. The Kurtosis feature belongs to the “global” category; the Homogeneity and Variance features belong to the “gray-level co-occurrence matrix” category; the GLN, ZSN, LZHGE, SZLGE, LZLGE and ZSV features belong to the “gray-level size zone matrix” category; and the Strength, Coarseness, Busyness, Complexity and Contrast features belong to the “neighborhood gray-tone difference matrix” category. Notably, the Variance and Contrast features could also originate from the “global” and “gray-level co-occurrence matrix” category, respectively. The “R” represents weights to bandpass sub-bands in wavelet filtering.

Abbreviations: Lasso, Least absolute shrinkage and selection operator; mRMR, Max-Relevance and Min-Redundancy; ID, identify number; sMRI, structural magnetic resonance imaging; L, left; R, right; Post, posterior; Sup, superior; Inf, inferior; Mid, middle; Orb, orbital; Ant, anterior; GLN, Gray-level nonuniformity; ZSN, Zone-size nonuniformity; LZHGE, Large zone high-gray-level emphasis; SZLGE, Small zone low gray-level emphasis; LZLGE, Large zone low-gray-level emphasis; ZSV, Zone-size variance; SZHGE, Small zone high gray-level emphasis; LGZE, Low gray-level zone emphasis; GLV, Gray-level variance; HGZE, High gray-level zone emphasis.

| **Supplementary Table 3. Classification performance of the SVM models (Linear and Sigmoid kernel)** | | | | | | | | |
| --- | --- | --- | --- | --- | --- | --- | --- | --- |
|  |  | **SVM model (kernel: linear)** | | |  | **SVM model (kernel: sigmoid)** | | |
| **Group** | **Method** | **ACC** | **SEN** | **SPE** |  | **ACC** | **SEN** | **SPE** |
| **Validation dataset** | Fisher score | 89.65%±5.09% | 87.06%±9.01% | 91.75%±6.58% |  | 90.92%±4.69% | 88.19%±7.80% | 93.18%±6.02% |
| **Test dataset** | Fisher score | 81.86%±6.86% | 77.26%±13.84% | 86.11%±7.82% |  | 85.17%±4.84% | 85.50%±10.94% | 84.86%±6.57% |
| **Validation dataset** | Lasso | 95.84%±3.22% | 93.68%±6.23% | 97.57%±3.40% |  | 95.86%±3.29% | 92.65%±6.64% | 98.31%±2.84% |
| **Test dataset** | Lasso | 89.10%±4.64% | 82.86%±8.95% | 94.87%±4.50% |  | 88.31%±5.68% | 79.51%±11.57% | 96.46%±4.05% |
| **Validation dataset** | mRMR | 92.34%±4.70% | 90.20%±8.45% | 94.15%±5.42% |  | 93.20%±4.17% | 89.20%±7.58% | 96.35%±4.39% |
| **Test dataset** | mRMR | 81.98%±6.21% | 74.42%±12.43% | 88.97%±6.44% |  | 84.87%±6.40% | 78.10%±13.73% | 91.13%±6.22% |

Supplementary Table 3. Classification performance of the SVM models (Linear and Sigmoid kernel)

Under the sample disturbance of five-fold cross-validation, we carried out three different kinds of composite function disturbances separately to screen features in the training dataset and repeated the process 100 times. The retained features were incorporated into the SVM model and RF model each time, and we then calculated the models’ classification performance in the validation dataset and test dataset separately. The measures are presented as mean ± standard deviation.

Abbreviations: SVM, support vector machine; ACC, accuracy; SEN, sensitivity; SPE, specificity; Lasso, Least absolute shrinkage and selection operator; mRMR, Max-Relevance and Min-Redundancy.

| \| **Supplementary Table 4. Classification performance of the SVM and RF models based on clinical features** \| \| \| \| \| \| \| \| \| --- \| --- \| --- \| --- \| --- \| --- \| --- \| --- \| \|  \| **SVM model (kernel: radial basis)** \| \| \|  \| **SVM model (kernel: linear)** \| \| \| \| **Group** \| **ACC** \| **SEN** \| **SPE** \|  \| **ACC** \| **SEN** \| **SPE** \| \| **Training dataset** \| 56.09%±2.07% \| 0.13%±2.87% \| 99.92%±1.69% \|  \| 56.09%±2.07% \| 0.13%±2.87% \| 99.92%±1.69% \| \| **Validation dataset** \| 56.01%±8.19% \| 0.20%±4.47% \| 99.89%±2.56% \|  \| 56.01%±8.19% \| 0.20%±4.47% \| 99.89%±2.56% \| \|  \| **SVM model (kernel: sigmoid)** \| \| \|  \| **RF model** \| \| \| \| **Group** \| **ACC** \| **SEN** \| **SPE** \|  \| **ACC** \| **SEN** \| **SPE** \| \| **Training dataset** \| 56.08%±2.05% \| 0.11%±2.45% \| 99.94%±1.43% \|  \| 99.73%±9.06% \| 99.44%±15.24% \| 99.95%±12.81% \| \| **Validation dataset** \| 56.00%±8.15% \| 0.08%±1.79% \| 99.91%±1.92% \|  \| 55.93%±0.51% \| 38.55%±1.11% \| 71.01%±0.31% \|   Supplementary Table 4. Classification performance of the SVM and RF models based on clinical features  Under the sample disturbance of five-fold cross-validation, the cohort 1 was randomly divided into a training set (80%) and a validation set (20%), we established the SVM and RF classification models based on their clinical information purely of the training date set, and then verified the classification performance on the validation set. The above steps were repeated 100 times, and the measures are presented as mean ± standard deviation.  Abbreviations: SVM, support vector machine; RF, random forest; ACC, accuracy; SEN, sensitivity; SPE, specificity.  **Supplementary Table 5. Traditional imaging indices of participants** | | | | | | |
| --- | --- | --- | --- | --- | --- | --- | --- | --- | --- | --- | --- | --- | --- | --- | --- | --- | --- | --- | --- | --- | --- | --- | --- | --- | --- | --- | --- | --- | --- | --- | --- | --- | --- | --- | --- | --- | --- | --- | --- | --- | --- | --- | --- | --- | --- | --- | --- | --- | --- | --- | --- | --- | --- | --- | --- | --- | --- | --- | --- | --- | --- | --- | --- | --- | --- | --- | --- | --- | --- | --- | --- | --- | --- | --- | --- | --- | --- | --- |
|  | **Cohort 1** | |  | **Cohort 2** | |  |
| **Group** | **Aβ_P (n = 78)** | **Aβ_N (n = 105)** | ***P* value** | **Cog_D (n = 24)** | **Cog_M (n = 27)** | ***P* value** |
| **ALFF** | 0.081±0.042 | 0.076±0.035 | 0.350 | 0.058±0.054 | 0.069±0.047 | 0.465 |
| **fALFF** | 0.084±0.063 | 0.085±0.053 | 0.905 | 0.082±0.049 | 0.071±0.052 | 0.443 |
| **ReHo** | 0.123±0.080 | 0.134±0.081 | 0.380 | 0.176±0.053 | 0.127±0.078 | 0.012^*^ |
| **Hip_volume (cm³)** | 3.10±0.34 | 3.08±0.32 | 0.719 | 2.69±0.36 | 3.03±0.28 | <0.0001^**^ |
| **BF_volume (cm³)** | 0.66±0.06 | 0.66±0.07 | 0.929 | 0.60±0.07 | 0.64±0.07 | 0.022^*^ |
| **Ent_volume (cm³)** | 1.94±0.21 | 1.94±0.24 | 0.968 | 1.78±0.25 | 1.93±0.23 | 0.023^*^ |
| **TI_volume (cm³)** | 1381.2±137.0 | 1346.4±130.3 | 0.082 | 1383.7±157.0 | 1362.8±113.2 | 0.585 |

Supplementary Table 5. Traditional imaging indices of participants

Cohort 1 was qualitatively divided into Aβ-P and Aβ-N groups according to the SUVR of participants (the cutoff was 1.18); cohort 2 was classified by the future cognitive outcomes of participants, including Cog-D and Cog-M groups. At baseline, all participants were cognitively healthy, and we made comparisons of traditional conventional indicators of AD (volumes of typical regions, ALFF, fALFF and ReHo of DMN) between the two groups of cohorts 1 and 2. Continuous measures are presented as mean ± standard deviation. Statistical analysis was conducted by independent two-sample two-tailed *t*-test for quantitative variables. * means *p*<0.05; ** means *p*<0.001.

Abbreviations: Aβ, amyloid-β; P, positive; N, negative; Cog, cognition; D, deteriorated; M, maintained; M, male; F, female; ALFF, amplitude of low frequency fluctuation; fALFF, fractional ALFF; ReHo, regional homogeneity; Hip, hippocampi; BF, basal forebrain; Ent, entorhinal cortex; TI, Total intracranial; DMN, default mode network; AD, Alzheimer’s disease.

Supplementary Figure 1. The recruitment process of participants

In cohort 1, we first screened the integrity of imaging data of participants from the SILCODE project, and then excluded a great number of individuals without amyloid-PET. The remaining received a complete battery of neuropsychological tests, including the evaluation of global cognition, different domains, anxiety, depression, living ability and others. Finally, 183 cognitively healthy participants were included and used as the training and validation dataset (**A, purple frames**).

In cohort 2, additional 51 cognitively healthy participants were evaluated by the same procedure while without amyloid PET examination, they were retrospectively selected and divided according to their prospective cognition outcomes. Their baseline data were used as an independent test dataset. Furthermore, the 24 ‘converters’ performed an extra MPMRI when they were found cognitive impairment (**B, wathet frames**).

Abbreviations: PET, positron emission tomography; AVLT, auditory verbal learning test; STT, shape trails test; AFT, animal fluency test; BNT, Boston naming test; MES, memory and execution screening; MMSE, mini-mental state examination; MoCA-B, Montreal cognitive assessment-basic; FAQ, Functional activities questionnaire; ECog, everyday cognition; CDR, clinical dementia rating; HAMD, Hamilton depression scale; HAMA, Hamilton anxiety scale; GDS, geriatric depression scale; PSQI, Pittsburgh sleep quality index; MCI, mild cognitive impairment; NIA-AA, National Institute of Aging-Alzheimer's Association; MPMRI, multi-parametric magnetic resonance imaging.

Supplementary Figure 2. The ROC curves of retained features and correlation analysis

The LZHGE feature of the left posterior cingulate gyrus on sMRI (ID: 6486) also had a high discriminating power in the test dataset, AUCs = 0.739 **(A)**. **(B)** shows the correlation of levels of this feature and mean cortical SUVR values in participants of cohort 1. The AUCs value increased to 0.863 when combined it with the stable high-frequency features **(C)**.

Abbreviations: LZHGE, Large zone high-gray-level emphasis; SUVR, standardized uptake value ratio; ROC, receiver operating characteristic; AUCs, areas under curve; sMRI, structural magnetic resonance imaging; Num, number.

Supplementary Figure 3. The adjusted correlation analysis

We made correlation analysis of the stable high-frequency features and the feature 6486, separately, after adjusting for age, gender, education, and Montreal Cognitive Assessment score. The features were the Coarseness feature of the left posterior cingulate gyrus on sMRI (ID: 6489) **(A)**, the LZHGE feature of the right posterior cingulate gyrus on sMRI (ID: 11517) **(B)**, the Variance feature of the left superior parietal gyrus on sMRI (ID: 27442) **(C)** and the LZHGE feature of the left posterior cingulate gyrus on sMRI (ID: 6486) **(D)**.

Supplementary Figure 4. Longitudinal change and survival analysis of the feature 6486

In the longitudinal researches, 37 participants were included, their cognition was normal at baseline and impaired during follow-up, with two progressing to dementia and 35 to mild cognitive impairment. As shown in **(A)** and **(B)**, the feature 6486 levels did not change at the two time points of different cognitive stages (*p*=0.1202) (two-tailed, *p*<0.05). Furthermore, these individuals were stratified into high-level (n=18) and low-level (n=19) groups by the baseline median level; **(C)** show Kaplan-Meier curves demonstrating the cumulative probabilities of conversion of the two groups (shaded area was the 95% confidence interval); differences are displayed by making log-rank tests (*p*<0.05), and there was no difference of the conversion time (*p*=0.44). The feature 6486 was the LZHGE feature of the left posterior cingulate gyrus on sMRI.

Abbreviations: NC, normal control; CI, cognitive impairment; sMRI, structural magnetic resonance imaging; LZHGE, Large zone high-gray-level emphasis.

**REFERENCES**

Aide N, Fruchart C, Nganoa C, Gac AC, Lasnon C (2020) Baseline 18F-FDG PET radiomic features as predictors of 2-year event-free survival in diffuse large B cell lymphomas treated with immunochemotherapy. *Eur Radiol* **30**, 4623-4632.

Amadasun M, King R (1989) Textural features corresponding to textural properties. *IEEE Transactions Systems Man & Cybernetics* **19**, 1264-1274.

Bondi MW, Edmonds EC, Jak AJ, Clark LR, Delano-Wood L, McDonald CR, Nation DA, Libon DJ, Au R, Galasko D, Salmon DP (2014) Neuropsychological criteria for mild cognitive impairment improves diagnostic precision, biomarker associations, and progression rates. *J Alzheimers Dis* **42**, 275-289.

Chandrashekar G, Sahin F (2014) A survey on feature selection methods. *Computers & Electrical Engineering* **1**, 16-28.

Chen G, Yang K, Du W, Hu X, Han Y (2019) Clinical Characteristics in Subjective Cognitive Decline with and without Worry: Baseline Investigation of the SILCODE Study. *J Alzheimers Dis* **72**, 443-454.

Chen KL, Xu Y, Chu AQ, Ding D, Liang XN, Nasreddine ZS, Dong Q, Hong Z, Zhao QH, Guo QH (2016) Validation of the Chinese Version of Montreal Cognitive Assessment Basic for Screening Mild Cognitive Impairment. *J Am Geriatr Soc* **64**, e285-285e290.

Cheng NM, Fang YH, Chang JT, Huang CG, Tsan DL, Ng SH, Wang HM, Lin CY, Liao CT, Yen TC (2013) Textural features of pretreatment 18F-FDG PET/CT images: prognostic significance in patients with advanced T-stage oropharyngeal squamous cell carcinoma. *J Nucl Med* **54**, 1703-1709.

Chu A, Sehgal CM, Greenleaf JF (1990) Use of gray value distribution of run lengths for texture analysis. *Pattern Recognit Lett* **11**, 415-419.

Dasarathy BV, Holder EB (1991) Image characterizations based on joint gray level—run length distributions. *Pattern Recognit Lett* **12**, 497-502.

HARALICK RM (1973) Textural features for image classification. IEEE Transaction on Systems, Man, and Cybernetics. *SMC* **3** .

Hong YJ, Park KW, Kang DY, Lee JH (2019) Prediction of Alzheimer's Pathological Changes in Subjective Cognitive Decline Using the Self-report Questionnaire and Neuroimaging Biomarkers. *Dement Neurocogn Disord* **18**, 19-29.

Huang M, Lu S, Yu L, Li L, Zhang P, Hu J, Zhou W, Hu S, Wei N, Huang J, Weng J, Xu Y (2017) Altered fractional amplitude of low frequency fluctuation associated with cognitive dysfunction in first-episode drug-naïve major depressive disorder patients. *BMC Psychiatry* **17**, 11.

Jack, C. R. Jr, Bennett, D. A., Blennow, K., Carrillo, M. C., Dunn, B., Haeberlein, S. B., et al. (2018). NIA-AA Research Framework: Toward a biological definition of Alzheimer's disease. *Alzheimers Dement* 14, 535-562. doi: 10.1016/j.jalz.2018.02.018.

Kang DW, Choi WH, Jung WS, Um YH, Lee CU, Lim HK (2017) Impact of Amyloid Burden on Regional Functional Synchronization in the Cognitively Normal Older Adults. *Sci Rep* **7**, 14690.

Li X, Wang X, Su L, Hu X, Han Y. Sino Longitudinal Study on Cognitive Decline (SILCODE): protocol for a Chinese longitudinal observational study to develop risk prediction models of conversion to mild cognitive impairment in individuals with subjective cognitive decline. BMJ Open. 2019. 9(7): e028188.

Lu J, Li D, Li F, Zhou A, Wang F, Zuo X, Jia XF, Song H, Jia J (2011) Montreal cognitive assessment in detecting cognitive impairment in Chinese elderly individuals: a population-based study. *J Geriatr Psychiatry Neurol* **24**, 184-190.

Mary, M., Galloway (1975) Texture analysis using gray level run lengths. *Computer Graphics and Image Processing* .

McKhann GM, Knopman DS, Chertkow H, Hyman BT, Jack CR Jr, Kawas CH, Klunk WE, Koroshetz WJ, Manly JJ, Mayeux R, Mohs RC, Morris JC, Rossor MN, Scheltens P, Carrillo MC, Thies B, Weintraub S, Phelps CH (2011) The diagnosis of dementia due to Alzheimer's disease: recommendations from the National Institute on Aging-Alzheimer's Association workgroups on diagnostic guidelines for Alzheimer's disease. *Alzheimers Dement* **7**, 263-269.

Pantic I, Jeremic R, Dacic S, Pekovic S, Pantic S, Djelic M, Vitic Z, Brkic P, Brodski C (2020) Gray-Level Co-Occurrence Matrix Analysis of Granule Neurons of the Hippocampal Dentate Gyrus Following Cortical Injury. *Microsc Microanal* **26**, 166-172.

Perrotin A, de Flores R, Lamberton F, Poisnel G, La Joie R, de la Sayette V, Mézenge F, Tomadesso C, Landeau B, Desgranges B, Chételat G (2015) Hippocampal Subfield Volumetry and 3D Surface Mapping in Subjective Cognitive Decline. *J Alzheimers Dis* **48 Suppl 1**, S141-150.

Perrotin A, La Joie R, de La Sayette V, Barré L, Mézenge F, Mutlu J, Guilloteau D, Egret S, Eustache F, Chételat G (2017) Subjective cognitive decline in cognitively normal elders from the community or from a memory clinic: Differential affective and imaging correlates. *Alzheimers Dement* **13**, 550-560.

Petersen RC (2004) Mild cognitive impairment as a diagnostic entity. *J Intern Med* **256**, 183-194.

Rizzo S, Botta F, Raimondi S, Origgi D, Fanciullo C, Morganti AG, Bellomi M (2018) Radiomics: the facts and the challenges of image analysis. *Eur Radiol Exp* **2**, 36.

Thibault G, Fertil B, Navarro C, Pereira S, Mari JL (2009) Texture Indexes and Gray Level Size Zone Matrix Application to Cell Nuclei Classification .

Vallières M, Freeman CR, Skamene SR, El Naqa I (2015) A radiomics model from joint FDG-PET and MRI texture features for the prediction of lung metastases in soft-tissue sarcomas of the extremities. *Phys Med Biol* **60**, 5471-5496.

Xia W, Chen Y, Zhang R, Yan Z, Zhou X, Zhang B, Gao X (2018) Radiogenomics of hepatocellular carcinoma: multiregion analysis-based identification of prognostic imaging biomarkers by integrating gene data-a preliminary study. *Phys Med Biol* **63**, 035044.
